# In the Eye of the Beholder: Challenge and Hindrance Appraisals of Work Characteristics and Their Implications for Employee’s Well-Being

**DOI:** 10.3389/fpsyg.2021.708309

**Published:** 2021-09-07

**Authors:** Peikai Li, Maria C. W. Peeters, Toon W. Taris, Yejun Zhang

**Affiliations:** ^1^Social, Health and Organisational Psychology, Utrecht University, Utrecht, Netherlands; ^2^Human Performance Management Group, Eindhoven University of Technology, Eindhoven, Netherlands; ^3^Department of Management, Robert C. Vackar College of Business & Entrepreneurship, University of Texas Rio Grande Valley, Edinburg, TX, United States

**Keywords:** challenge appraisal, employee well-being, hindrance appraisal, job demands, job resources

## Abstract

Previous research on the association between job characteristics and employee well-being has returned mixed results. In particular, the possible impact of individual appraisal of these job characteristics has not been well-acknowledged. To address this limitation, we drew on appraisal theory and examined: (a) how workers appraise particular job characteristics, and (b) how these appraisals affect the relationships between these job characteristics and well-being (i.e., work engagement and burnout). We tested our hypotheses across two studies. In a cross-occupation sample (Study 1, *n* = 514), we found that job demands and resources can be appraised as both challenges and hindrances. In addition, challenge appraisals can mitigate the detrimental impact of job demands on engagement and burnout; and hindrance appraisals can strengthen the detrimental effects of job demands on burnout. Further, hindrance appraisals of job resources reduce their beneficial effects on engagement and burnout. Study 2 (*n* = 316 nurses in a hospital) further showed that challenge appraisals of job demands can reduce their impact on burnout while challenge appraisals of job resources will strengthen their positive effect on employee engagement and burnout. We discuss study implications as well as future research directions.

## Introduction

Although scholars have often classified job characteristics as either job demands or job resources (e.g., Demerouti et al., 2001), this distinction has not remained unchallenged. Drawing on stress research, organizational researchers have expanded traditional job characteristics theory (e.g., the JD-R model, Demerouti et al., 2001; the Job Demand-Control model, Karasek, 1979) by re-categorizing job demands as either challenge or hindrance demands (e.g., van den Broeck et al., 2010; Teng et al., 2020). Although this distinction has certainly advanced our understanding of how different types of demands relate to important organizational and individual outcomes, the role of employees’ subjective appraisals of their job characteristics has not yet been well-acknowledged and needs further investigation (Parker, 2014; González-Morales and Neves, 2015).

Appraisals are defined as an individual’s interpretation of particular job characteristics as having the potential for personal gain and growth (challenges) or as constraints (hindrances; Cavanaugh et al., 2000; LePine et al., 2016). Building on the notion that individual functioning results from the interaction between individual and environmental factors (i.e., person-context interaction theory, Magnusson and Stattin, 1996), Li et al. (2020) demonstrated that demands can to some extent be appraised simultaneously as challenges and hindrances, and that individuals’ different appraisals can moderate the job demands–employee well-being relationships. However, as employees face not only job demands but also job resources in their work situation, it would seem that the effects of job resources on well-being may also be contingent upon individual appraisal. Building on this argument and recent empirical studies (e.g., Li et al., 2017, 2020), we propose that appraisals may influence the magnitude of the effects of job demands and job resources on employee well-being.

Our study advances the job characteristics literature by examining: (a) how employees appraise their job characteristics, and (b) whether and how these appraisals influence job characteristics–well-being relationships. First, instead of using an *a priori*-categorization of particular job characteristics (i.e., job demands and resources) as either challenges or hindrances (Bakker and Demerouti, 2017), we empirically test how employees appraise these job characteristics and how these appraisals affect their well-being. In doing so, we aim to extend the Job Demands-Resources model and the Challenge-Hindrance Stressor Framework by looking at the potential differentiated effects of the same job characteristics for different employees, and expand the appraisal literature by investigating how the appraisal of resources is related to well-being in contrast to the predominant focus on the appraisal of job demands in previous research. Second, appraisal-based studies have predominantly taken appraisal as a mediating variable in the job characteristics–outcomes relationships (e.g., Ohly and Fritz, 2010; Espedido and Searle, 2018; Liu and Li, 2018). We extend this research by testing how individual differences in appraisals influence the degree to which employees react to their job demands and job resources. This also responds to O’Brien and Beehr’s (2019) argument that “appraisals could be moderators, although little research has reported on that possibility” (p. 6). Finally, our study advances previous research by investigating both challenge and hindrance appraisals of job characteristics. This is important, as both types of appraisals can occur simultaneously with regard to a situational demand (Folkman, 1984; Gilboa et al., 2008).

### Challenge and Hindrance Job Characteristics

The Job Demands-Resources (JD-R) model (Demerouti et al., 2001) divides work characteristics into two categories: job demands and job resources (Bakker and Demerouti, 2017). Job demands are defined as the physical, psychological, social, or organizational aspects of the job that require sustained physical and/or psychological (cognitive and emotional) effort and that are therefore associated with certain physiological and/or psychological costs (Bakker et al., 2004). Examples are administrative hassles, emotional conflict, and role overload (Nahrgang et al., 2011). Job resources refer to the physical, psychological, social, or organizational aspects of the job that are functional in achieving work goals and/or that stimulate personal growth and development (Bakker et al., 2004). Examples include job autonomy, social support, and coworker support (Crawford et al., 2010).

Although previous studies have explored the relationships between job demands and resources and their outcomes (Bakker and Demerouti, 2017), not all findings are consistent with the hypothesized relationships (Olafsen et al., 2018). For example, Bakker et al. (2003) found that workload was positively rather than negatively associated with dedication (cf. Van Den Broeck et al., 2008). Similarly, in a longitudinal study, Mauno et al. (2007) reported that time demands were positively related to absorption. These findings suggest a need to revisit the relevant theories and examine the possible moderators that might alter these relationships. In addition, empirical studies showed that an excess of autonomy seems to have negative effects on employee well-being (Wielenga-Meijer et al., 2011; Stiglbauer and Kovacs, 2018). Consequently, researchers have begun to argue that: (a) not all demands are created equal, and (b) job resources can have detrimental effects as well (Wielenga-Meijer et al., 2011; Stiglbauer and Kovacs, 2018).

The discussion regarding the inconsistent effects of job demands currently mainly occurs within the challenge-hindrance demands framework (Cavanaugh et al., 2000; Olafsen et al., 2018). *Challenge demands* are defined as job demands that require efforts but that also present the potential for personal growth and rewards (e.g., workload, time pressure, and job complexity). *Hindrance demands* refer to job demands that interfere with or inhibit an individual’s ability to achieve valued goals and that thwart growth and gains (e.g., role conflict, role ambiguity, and organizational constraints). Meta-analytical reviews have supported the assumption that hindrance demands are associated with negative outcomes such as higher turnover and withdrawal behavior, whereas challenge demands are positively related to more desirable employee attitudes (e.g., higher job satisfaction, organizational commitment, and lower turnover intentions), job performance (LePine et al., 2005; Podsakoff et al., 2007), and safety outcomes (Clarke, 2012).

An alternative explanation for the inconclusive effects of job demands on outcomes draws on the idea that individual appraisal may be relevant as well. An appraisal-based approach assesses why some employees perceive a particular demand as a challenge, whereas others perceive the same demand as a hindrance. Moreover, it also allows for the fact that some demands can be perceived concurrently as challenging and hindering. For example, Webster et al. (2011) reported that workers perceived job demands such as workload, responsibility, role conflict, and role ambiguity concurrently as challenges and hindrances. In a related vein, Searle and Auton (2015) found that workers appraised time pressure as a challenge to the same degree as a hindrance. In summary, several empirical studies support the merits of including appraisals of job demands in work psychological research by demonstrating that these appraisals consistently explain unique variance in a study’s outcome variables (e.g., creative performance, Li et al., 2018; affect, Searle and Auton, 2015). Thus, it is imperative to extend current research to consider the role of appraisals on the effects of job characteristics.

### The Role of Cognitive Appraisals of Job Characteristics

According to lazarus and Folkman’s (1984) transactional theory of stress, one’s response to a stressful event depends on how one appraises the situation. In the primary appraisal stage, a person will evaluate how stressful the situation is. In the secondary stage of the appraisal – which occurs almost at the same time – people will evaluate what, if anything, can be done to overcome or to prevent harm, or to improve the prospects for benefit (Folkman et al., 1986). A person usually evaluates a situation based on how much is at stake and how controllable the situation is. If a situation is seen as a *challenge*, it will be viewed as taxing, but also as providing opportunities for personal gains, such as mastery, learning, or personal growth. Challenge appraisal thus indicates that with effort, the job characteristics can be mastered (Skinner and Brewer, 2002). Conversely, *hindrance appraisals* are defined as an individual’s subjective interpretation that job characteristics have the potential to interfere with or thwart an individual’s attempt to achieve valued goals (Cavanaugh et al., 2000; Searle and Auton, 2015). The transactional theory of stress further denotes that primary appraisal is an essential way in which an individual assesses the meaning and the significance of the situation, and as a major psychological process that connects stressors to outcomes. In addition to the degree to which people evaluate their situation as a challenge and/or hindrance, the transactional theory of stress also contends that primary appraisal impacts the valence of outcomes an individual will experience, such as strain, well-being, motivation, and performance (lazarus and Folkman, 1984; LePine et al., 2005).

### Appraisal of Job Demands as a Boundary Condition

Following the transactional theory of stress (lazarus and Folkman, 1984), studies on the appraisal of job characteristics usually treat appraisal as a mediator (Boswell et al., 2004; Tuckey et al., 2015; Liu and Li, 2018; Mitchell et al., 2019; Sessions et al., 2019). However, O’Brien and Beehr (2019) pointed out that “appraisals could be moderators” (p. 6). We propose that appraisals can also serve as a moderator. Work in general is taxing on personal resources (Demerouti et al., 2001), but if workers appraise a particularly demanding situation as something that can be overcome and that may lead to growth and rewards, the presumed detrimental effect on employee well-being will be weaker (Li et al., 2020). On one hand, such a challenge appraisal contributes to employee motivation in dealing with job demands (e.g., Liu and Li, 2018). On the other hand, high challenge appraisal has been established as adaptive in dealing with stressful events, as it is associated with more confident coping expectancies and more beneficial perceptions of stressful events (Skinner and Brewer, 2002). As a result, high challenge appraisal may buffer the detrimental effects of job demands.

In addition, previous studies have shown that high job demands are associated with increases in burnout (e.g., the JD-R model, Demerouti et al., 2001) and decreases in work engagement (e.g., Hu et al., 2017). Thus, we expect that job demands (i.e., time urgency, role conflict and emotional demands) will be positively related to burnout and negatively to engagement. These demands were chosen because Alarcon’s (2011) meta-analysis showed that they are well-established and important job demands in relation to employee well-being. Further, these demands are not consistently categorized as a challenge or a hindrance (e.g., Crawford et al., 2010; Bakker and Sanz-Vergel, 2013; Zhang et al., 2013; Albrecht, 2015; Baethge et al., 2019; Mazzola and Disselhorst, 2019). We expect a negative link between these demands and work engagement and a positive relationship between them and burnout.

*Hypothesis 1:* Job demands (i.e., time urgency, role conflict and emotional demands) will be positively related to burnout and negatively related to engagement.

Building on the transactional theory of stress (lazarus and Folkman, 1984) and empirical evidence (e.g., Li et al., 2017), we propose that individual differences in appraisals are likely to affect how employees deal with their job demands and, thus, the effects of exposure to these job demands. This theory suggests that appraisal is essential as it determines a person’s perception of the meaning and significance of stressful events for his/her well-being, as well as to what extent a situation can be changed or accepted (lazarus and Folkman, 1984). In particular, information about appraisals will determine how one will attempt to cope with stressful situations. Coping refers to a process in which individuals “constantly change cognitive and behavioral efforts to manage specific external and/or internal demands” (lazarus and Folkman, 1984, p. 141). Two types of coping exist: (a) avoidance-oriented coping (i.e., avoiding thinking about the job demands or distancing oneself from the demands required), and (b) problem-focused coping (e.g., deliberate efforts to solve the problem or efforts to change the situation). When a hindering environmental condition is perceived as if nothing can be done to change it, avoidance-focused coping is more to occur. Conversely, if a situation is appraised as amenable to change, problem-focused coping is more likely (lazarus and Folkman, 1984). Since job demands can be appraised as both challenges and hindrances (Folkman, 1984; Webster et al., 2011; Searle and Auton, 2015), for workers who perceive a particular job demand as something that is controllable and can be overcome and that may lead to growth and rewards, employees are more likely to employ a problem-focused coping strategy. Thus, the assumed adverse effects of this demand on burnout and engagement will be relatively small. In contrast, if workers appraise a particular demand as a hindrance, the potential for constraints and thwarted growth will lead them to adopt an avoidance-oriented approach (lazarus and Folkman, 1984) and to experience stress, and this would magnify the hypothesized adverse effects of this demand. Therefore, we expect that:

*Hypothesis 2*: Challenge appraisal moderates the negative relationships between job demands (i.e., time urgency, role conflict and emotional demands) and engagement, such that these relationships are weaker when challenge appraisal is high.

*Hypothesis 3*: Challenge appraisal moderates the positive relationships between job demands (i.e., time urgency, role conflict and emotional demands) and burnout, such that these relationships are weaker when challenge appraisal is high.

*Hypothesis 4*: Hindrance appraisal moderates the negative relationships between job demands (i.e., time urgency, role conflict and emotional demands) and engagement, such that these relationships are stronger when hindrance appraisal is high.

*Hypothesis 5*: Hindrance appraisal moderates the positive relationships between job demands (i.e., time urgency, role conflict and emotional demands) and burnout, such that these relationships are stronger when hindrance appraisal is high.

### Appraisal of Job Resources as a Boundary Condition

Based on the JD-R model, job resources are expected to lead to desirable outcomes (e.g., higher engagement and lower burnout); however, some theoretical perspectives suggest that high levels of job resources might backfire. Both Warr’s vitamin model (which stipulates non-linear relationships between job characteristics and employee well-being; Warr, 1987) and person-environment (PE) fit theory (Edwards, 1991) suggest that negative outcomes may result from an excessive amount of some job resources. If environmental resources are not compatible with employees’ standards, employees will experience misfits and, consequently, a decrease in their well-being and outcomes (Edwards, 1991; van Vianen, 2018). For example, Wielenga-Meijer et al. (2011) found that increases in autonomy may have detrimental effects on learning outcomes. Similarly, experimental studies found that social support can also elicit negative reactions (Deelstra et al., 2003). A theoretically possible reason for the detrimental effect of resources draws on how employees appraise their resources. For instance, receiving instrumental social support at work will sometimes have an undesirable effect as it triggers feelings of inferiority and incompetence, which threats one’s self-esteem (Fisher et al., 1982). As Wielenga-Meijer et al. (2011) argued, the reason why autonomy fosters people’s motivation to learn is possibly that it leads to increased levels of *challenge*, which implies that resources can be appraised differently by employees.

In line with these findings on the cognitive appraisal of job demands, employees may experience job resources to some degree as both a challenge and/or a hindrance (Schaufeli and Taris, 2014). When an employee experiences a lack of resources, this might imply that they must spend more effort to achieve work goals. According to the JD-R model (Bakker and Demerouti, 2017), effort expenditure is a key characteristic of a job demand, which means that a lack of resources may also be construed as a job demand. Because job demands are perceived differently by workers (Webster et al., 2011; Searle and Auton, 2015), resources may be subject to similar individual variations in appraisals. Specifically, employees may perceive a particular job resource both as a challenge and a hindrance. For instance, social support can be appraised negatively (i.e., hindrance), as it may threaten one’s self-esteem (Fisher et al., 1982). However, it can also be appraised as a challenge, as it provides employees with resources (Bakker and Demerouti, 2017). On the other hand, previous studies have shown that exposure to job resources is a predictor of engagement (Bakker and Demerouti, 2017) and a decrease in burnout (e.g., Hu et al., 2017). In the current study, we chose autonomy, social support (of one’s colleagues and supervisor), and feedback from others as typical job resources. These resources were selected because meta-analytic reviews have shown that these are well-established resources that predict burnout and work engagement (Christian and Slaughter, 2007). Therefore, based on theoretical arguments (e.g., the JD-R model, Demerouti et al., 2001) as well as empirical research (e.g., Hu et al., 2017), we propose that:

*Hypothesis 6:* Job resources (i.e., autonomy, colleague support, supervisor support, feedback) will be positively related to work engagement and negatively related to burnout.

Further, we argue that the magnitude of the job resources–well-being relationship will vary as a function of appraisal. Although work is taxing on personal resources, individuals with high job resources are better able to cope with their work-related demands than others (Schaufeli and Taris, 2014). Thus, appraising resources as challenging and allowing for potential growth and opportunities will have more beneficial effects on employee well-being than seeing such resources as hindering. Conversely, seeing a job resource as a hindrance and focusing on its potential constraints may have detrimental effects on its associations with outcomes (e.g., Fisher et al., 1982). For example, high levels of autonomy are likely to turn into “unavoidable requirements” in that this could create a seemingly intractable information problem, meaning that it is hard to gather information and take decisions (e.g., Schwartz et al., 2002). Thus, we propose that seeing a job resource as a hindrance (seeing its *gain as pain*), the potential for constraints will lead employees to be reluctant in adopting an approach-oriented coping strategy (lazarus and Folkman, 1984), which will undermine the motivational effects of this resource. Therefore, we hypothesize that challenge and hindrance appraisals moderate the relationship between job resources and employee well-being.

*Hypothesis 7*: Challenge appraisal moderates the positive relationships between job resources (i.e., autonomy, colleague support, supervisor support, feedback) and engagement, such that these relationships are stronger when challenge appraisal is high.

*Hypothesis 8*: Challenge appraisal moderates the negative relationships between job resources (i.e., autonomy, colleague support, supervisor support, feedback) and burnout, such that these relationships are stronger when challenge appraisal is high.

*Hypothesis 9*: Hindrance appraisal moderates the positive relationships between job resources (i.e., autonomy, colleague support, supervisor support, feedback) and engagement, such that these relationships are weaker when hindrance appraisal is high.

*Hypothesis 10*: Hindrance appraisal moderates the negative relationships between job resources (i.e., autonomy, colleague support, supervisor support, feedback) and burnout, such that these relationships are weaker when hindrance appraisal is high.

### Overview of Studies

We conducted two studies to test our hypotheses. In Study 1, we tested our hypotheses by asking for employees’ *general* appraisal of certain job characteristics in scenarios in a sample of working adults from multiple organizations from China. In Study 2, we aimed to replicate our findings in a sample of nurses from a single organization (i.e., all participants had similar working characteristics), where we measured appraisal by having these nurses assess their *own* job characteristics.

## Study 1 Method

### Procedures and Participants

The participants in this study were recruited through the online platform *SoJump*, which is similar to MTurk and Qualtrics. We sent participants an introductory email including a link to the online questionnaire. All participants (consisting of employees holding a full-time job in a broad variety of occupations) joined voluntarily and they were assured that their responses would stay anonymous. We sent the questionnaires to 2,611 Chinese employees and received 525 completed questionnaires in return (overall response rate of 20.11%). As a reward for completing the survey, participants received the equivalent of €1.67 in Chinese RMB. Eleven participants were deleted based on their response times, which showed that they completed the survey in a period over three standard deviations longer than the sample mean time (Curran, 2016). This resulted in a final sample of 514 participants. The average age of these participants was 33.77 years and the average organizational tenure was 7.30 years. There were 292 women (56.8%) in the sample, and participants worked on average 42.29 h a week. Most of the participants held a bachelor’s degree (73.2%).

### Measures

All questionnaires were in Chinese. Where applicable, we used scales that had already validated in the Chinese context. Otherwise, we followed a back-translation process to ensure semantic equivalence (Brislin et al., 1973). The original English items were first translated into Chinese by the first author and then translated back into English by two other researchers. Then together with two psychology professors, we compared the English and Chinese versions of the items to guarantee their accuracy, and if necessary we made modifications for some minor discrepancies. Unless otherwise indicated, items were scored on 7-point Likert scales (1 = *strongly disagree* and 7 = *strongly agree*).

### Job Demands

*Time urgency* was measured with four items (e.g., Rodell and Judge, 2009; Maruping et al., 2015). A sample item is “The amount of time provided to complete my tasks is short.” Cronbach’s alpha was α = 0.86. *Role conflict* was measured with three items from the Cross-Cultural Role Conflict, Ambiguity, and Overload Scale (Peterson et al., 1995). A sample item is “Different people quite often ask me to do the same thing in different ways.” Cronbach’s alpha was α = 0.84. *Emotional demands* were assessed with four items from the Emotional demands scale (Peeters et al., 2005). An example is “Does your work bring you in upsetting/disturbing situations?” (1 = *never* and 5 = *often*). Cronbach’s alpha was α = 0.76.

### Job Resources

*Colleague support* was measured with four items from Peeters et al. (1995). A sample item is “If needed, my colleagues help me with a certain task” (1 = *never* and 5 = *often*). Cronbach’s alpha was α = 0.65. For *supervisor support*, we used the same items but replaced “colleague” with “supervisor”. Cronbach’s alpha was α = 0.72. We used three items from the Work Design Questionnaire (WDQ, Morgeson and Humphrey, 2006) to measure *feedback from others*. An example item is “I receive a great deal of information from my manager and coworkers about my job performance.” Cronbach’s alpha for this scale was 0.65. Two items from the WDQ were used to assess *autonomy*, including “The job provides me with significant autonomy in making decisions.” Cronbach’s alpha was α = 0.81.

### Appraisals of Demands and Resources

To measure appraisals, we used the Challenge and Hindrance Appraisals scale (Searle and Auton, 2015). The challenge and hindrance appraisals of each demand and resource were measured separately. Specifically, for each of the three demands and four resources included in our study, participants were asked to indicate to what extent they considered this specific job characteristic as a challenge or a hindrance. For each job characteristic, challenge and hindrance appraisals were measured using two separate four-item scales. In the introduction of these challenge/hindrance scales, the items tapping the job characteristic to be appraised were included in a slightly rephrased form. Taking feedback from others as an example, the introduction reads “Imagine the following situation: Chris says ‘*on my job, I receive feedback on my performance from other people in my organization, and other people in the organization, such as managers and coworkers, provide information about the effectiveness (e.g., quality and quantity) of my job performance*.’” Then we asked participants “In general, I believe that having a job like Chris’s…”, which was followed by the two four-item sets tapping challenge appraisal (e.g., “… will make the experience educational”) and hindrance appraisal (e.g., “… will restrict my capabilities”). Similar scenarios were developed for the other job resources and demands. Cronbach’s alphas for the appraisals of job resources and demands ranged from 0.76 to 0.91 (see Table 2).

### Well-Being

*Work engagement* was assessed using nine items (e.g., “At my work, I feel bursting with energy”) from the Utrecht Work Engagement Scale (Schaufeli et al., 2006). Cronbach’s alpha was 0.93. *Burnout* was measured with nine items of the Chinese version (Hu and Schaufeli, 2011) of the Maslach Burnout Inventory-General Survey (MBI-GS, Maslach et al., 1986). Sample items are “I feel used up at the end of a workday” (0 = *never* and 6 = *every day*). Cronbach’s alpha for this scale was 0.92.

### Analytical Strategy

First, we conducted confirmatory factor analyses (CFA) to test the measurement model. We used the maximum likelihood estimation approach and conducted the analyses in Mplus (Muthén and Muthén, 1998-2011). We then tested the hypotheses using regressions in SPSS. To maintain adequate power for detecting effects (Cohen et al., 2013), we utilized a piecemeal approach and tested the moderation effects in separate models. To ease interpretation, we used centered variables when computing the interaction terms (Hayes, 2013). We further tested our hypotheses while controlling for social demographics (i.e., age, gender, education, tenure, work time, and industry). The pattern of the results did not change, supporting the robustness of the findings.

### Study 1 Results

#### CFA Results

We first conducted CFA to test the measurement model. In the first model, all items loaded on their corresponding hypothesized constructs. This 23-factor model yielded good fit statistics [χ^2^_(3232)_ = 5,459.85, *p* < 0.001; RMSEA = 0.04; CFI = 0.92; TLI = 0.91; SRMR = 0.04] against five alternative measurement models. The results are presented in Table 1. In addition, to examine the potential common method bias, we tested a model where an additional unmeasured latent method factor was included. The results showed that the common method factor explained 6.8% of the variance in the measurement items, so it did not impose an undue influence on our findings.

**TABLE 1:** Results of confirmatory factor analyses in Study 1.

**Model**	**χ^2^**	***df***	**RMSEA [90% CI]**	**CFI**	**TLI**	**SRMR**	**AIC**	**BIC**
23-factor parceled	5459.85	3232	0.04 [0.035,0.038]	0.92	0.91	0.04	129566.82	131721.87
23-factor not parceled	7616.54	4402	0.04 [0.036,0.039]	0.90	0.89	0.04	151167.62	153488.12
26 factor	7463.87	4330	0.04 [0.036,0.039]	0.90	0.89	0.04	151158.95	153784.89
11-factor	15065.95	3430	0.08 [0.08,0.083]	0.58	0.56	0.13	138776.91	140092.01
13-factor	9675.82	3407	0.06 [0.058,0.061]	0.77	0.76	0.06	133432.79	134845.46
8-factor	10741.25	3457	0.06 [0.063,0.065]	0.73	0.06	0.07	134398.22	135598.77
23-factor parceled with common method effects	5442.23	3231	0.04 [0.035,0.038]	0.92	0.91	0.04	129551.20	131710.49
1-factor	23108.92	3485	0.11 [0.103,0.106]	0.28	0.27	0.16	146709.89	147791.65

*RMSEA, root mean square error of approximation; CFI, comparative fit index; TLI, Tucker–Lewis index; SRMR, standardized root mean residual; CI, confidence interval; AIC, Akaike information criterion; BIC, Bayesian information criterion.*

*23-factor model-parceled: three demands: time urgency, role conflict, and emotional demands; four resources: autonomy, feedback, colleague support, and supervisor support; seven challenge and seven hindrance appraisals of demands and resources: and two outcomes with nine engagement items were mean-parceled as three indicators based on the three engagement dimensions and loading on one latent engagement factor; nine burnout items were mean-parceled as two indicators representing exhaustion and cynicism, and loading on one latent burnout factor);*

*23-factor not parceled: 23-factor model-parceled with nine items of engagement loaded on one, and nine items of burnout loaded on another latent factor.*

*26-factor: 23-factor not parceled with two outcomes loaded as five factors: vigor, dedication, absorption, exhaustion, and cynicism.*

*11-factor: 23-factor model-parceled with seven challenge appraisals into one, and seven hindrance appraisals loaded on one factor. 13-factor: 11-factor splitting appraisals as four factors: challenge/hindrance appraisals of demands and challenge/hindrance appraisals of resources.*

*8-factor: 11-factor combining three demands into one factor, and four resources into another factor.*

*23-factor parceled with common method effects: 23-factor model-parceled added a latent method factor allowing all items loaded on the method factor.*

*1-factor model: with all variables loaded onto one factor.*

#### Challenge and Hindrance Ratings of Job Characteristics

Table 2 presents the descriptive statistics, internal consistency reliabilities, and zero-order correlations of the study variables. This table shows that time urgency was more likely considered a challenge (*M* = 4.42, *SD* = 1.37) than a hindrance (*M* = 4.17, *SD* = 1.42; *T* = 2.43, *p* = 0.02). However, role conflict (*M*_challenge_ = 4.10, *SD* = 1.41; *M*_hindrance_ = 4.33, *SD* = 1.42; *T* = –2.17, *p* = 0.03) and emotional demands (*M*_challenge_ = 3.80, *SD* = 1.54; *M*_hindrance_ = 4.55, *SD* = 1.48; *T* = –6.53, *p* < 0.001) were more often perceived as a hindrance than as a challenge. As for job resources, the results in Table 2 show that autonomy was more likely considered a challenge (*M* = 5.53, *SD* = 0.91) than a hindrance (*M* = 2.92, *SD* = 1.45; *T* = 28.82, *p* < 0.001). Similar results were found for supervisor support (*M*_challenge_ = 5.35, *SD* = 0.95; *M*_hindrance_ = 3.23, *SD* = 1.42; *T* = 23.58, *p* < 0.001), feedback from others (*M*_challenge_ = 5.38, *SD* = 0.95; *M*_hindrance_ = 3.11, *SD* = 1.44; *T* = 26.2, *p* < 0.001), and colleague support (*M*_challenge_ = 5.26, *SD* = 0.98; *M*_hindrance_ = 3.24, *SD* = 1.49; *T* = 22.22, *p* < 0.001). In addition, the *SD*s of all appraisals were different from zero, with the average *SD*s being 1.44 for demands and 1.20 for resources (on a 7-point Likert scale). This demonstrates that employees are quite different in their appraisals of these job characteristics (see Table 2).

**TABLE 2:** Means, standard deviations, and correlations among the study variables in Study 1.

**Variable**	**Mean**	***SD***	**Skewness**	**Kurtosis**	**1**	**2**	**3**	**4**	**5**	**6**	**7**	**8**	**9**	**10**	**11**	**12**
(1) Age	33.77	7.14	1.27	1.83	1											
(2) Gender	1.57	0.50	–0.28	–1.93	−0.21**	1										
(3) Education	2.89	0.62	–0.78	2.43	−0.16**	0.01	1									
(4) Work time	42.69	10.25	–0.69	5.01	–0.05	–0.02	–0.05	1								
(5) Tenure	7.30	6.11	2.36	7.09	0.65**	−0.18**	–0.06	−0.10*	1							
(6) Time urgency	4.40	1.34	–0.36	–0.73	0.04	−0.09*	0.14**	0.12**	0.03	(0.86)						
(7) Role conflict	4.07	1.48	–0.24	–0.93	–0.05	–0.03	0.06	0.00	–0.04	0.53**	(0.84)					
(8) Emotional demand	2.90	0.77	0.05	–0.67	–0.06	0.07	0.02	0.05	−0.09*	0.49**	0.51**	(0.76)				
(9) Autonomy	4.68	1.48	–0.61	–0.42	–0.01	0.07	0.08	−0.13**	0.01	−0.21**	−0.21**	−0.25**	(0.81)			
(10) Colleague support	3.44	0.70	–0.39	0.09	–0.04	–0.02	0.02	–0.06	–0.01	−0.17**	−0.16**	−0.18**	0.20**	(0.65)		
(11) Supervisor support	3.29	0.75	–0.43	0.02	–0.02	0.01	0.05	−0.13**	0.01	−0.19**	−0.24**	−0.29**	0.41**	0.52**	(0.72)	
(12) Feedback	3.54	0.78	–0.54	0.03	0.00	–0.07	0.05	0.00	–0.01	–0.07	−0.16**	−0.17**	0.17**	0.48**	0.51**	(0.65)
(13) Time urgency CA	4.42	1.37	–0.51	–0.47	0.12**	−0.09*	0.08	−0.15**	0.15**	0.15**	0.13**	0.00	0.17**	0.02	0.03	0.03
(14) Time urgency HA	4.17	1.42	–0.26	–0.86	−0.10*	0.08	0.07	–0.02	–0.08	0.13**	0.10*	0.26**	–0.03	0.03	0.06	0.00
(15) Role conflict CA	4.10	1.41	–0.36	–0.74	0.18**	–0.04	0.05	−0.14**	0.18**	0.15**	0.19**	0.02	0.19**	0.03	0.08	0.05
(16) Role conflict HA	4.33	1.42	–0.36	–0.71	−0.12**	0.04	0.07	0.04	−0.13**	0.13**	0.06	0.22**	−0.10*	–0.02	−0.09*	–0.09
(17) Emotional demand CA	3.80	1.54	–0.04	–1.13	0.17**	−0.13**	0.05	−0.18**	0.19**	0.11*	0.11*	0.03	0.14**	0.03	0.03	0.01
(18) Emotional demand HA	4.55	1.48	–0.51	–0.69	−0.16**	0.12**	0.06	0.06	−0.12**	0.10*	0.07	0.15**	0.01	0.01	0.04	0.03
(19) Autonomy CA	5.53	0.91	–0.66	0.10	–0.08	0.05	0.09*	0.16**	–0.07	0.01	–0.07	–0.05	0.03	0.21**	0.18**	0.19**
(20) Autonomy HA	2.92	1.45	0.64	–0.70	0.07	–0.06	0.04	−0.28**	0.10*	0.18**	0.24**	0.25**	0.09	–0.08	–0.02	–0.08
(21) Colleague support CA	5.26	0.98	–0.75	0.67	–0.02	0.03	0.08	0.00	0.04	0.07	0.01	0.00	0.07	0.23**	0.16**	0.21**
(22) Colleague support HA	3.24	1.49	0.41	–0.93	0.00	–0.06	0.00	−0.17**	0.04	0.17**	0.18**	0.25**	0.04	−0.11*	–0.07	–0.08
(23) Supervisor support CA	5.35	0.95	–0.73	0.59	–0.06	0.04	0.13**	0.00	–0.03	0.02	–0.04	–0.07	0.12**	0.29**	0.24**	0.20**
(24) Supervisor support HA	3.23	1.42	0.30	–0.94	–0.02	–0.03	0.00	−0.16**	–0.01	0.15**	0.19**	0.24**	0.07	−0.09*	–0.07	−0.12**
(25) Feedback CA	5.38	0.95	–0.74	0.68	–0.01	0.04	0.13**	0.07	0.05	0.01	–0.08	–0.07	0.14**	0.24**	0.23**	0.25**
(26) Feedback HA	3.11	1.44	0.49	–0.83	0.01	–0.07	0.02	−0.19**	0.06	0.18**	0.25**	0.25**	0.07	–0.09	–0.08	−0.12**
(27) Burnout	3.66	1.19	0.01	–0.73	–0.06	–0.03	–0.01	0.05	–0.06	0.42**	0.453**	0.59**	−0.35**	−0.34**	−0.42**	−0.33**
(28) Engagement	4.29	1.16	–0.23	–0.39	0.02	–0.04	0.14**	−0.20**	0.07	−0.14**	−0.17**	−0.29**	0.39**	0.37**	0.48**	0.35**

**Variable**	**13**	**14**	**15**	**16**	**17**	**18**	**19**	**20**	**21**	**22**	**23**	**24**	**25**	**26**	**27**	**28**

(13) Time urgency CA	(0.87)															
(14) Time urgency HA	−0.43**	(0.87)														
(15) Role conflict CA	0.60**	−0.23**	(0.85)													
(16) Role conflict HA	−0.25**	0.53**	−0.46**	(0.88)												
(17) Emotional demand CA	0.56**	−0.16**	0.62**	−0.22**	(0.91)											
(18) Emotional demand HA	−0.22**	0.48**	−0.29**	0.46**	−0.51**	(0.91)										
(19) Autonomy CA	0.02	0.05	–0.06	0.08	–0.07	0.11*	(0.77)									
(20) Autonomy HA	0.28**	0.27**	0.34**	0.12**	0.43**	0.05	−0.48**	(0.91)								
(21) Colleague support CA	0.14**	0.05	0.04	0.10*	–0.02	0.19**	0.49**	−0.19**	(0.77)							
(22) Colleague support HA	0.21**	0.31**	0.31**	0.12**	0.40**	0.06	−0.26**	0.70**	−0.37**	(0.91)						
(23) Supervisor support CA	0.10*	0.06	–0.01	0.11**	–0.05	0.17**	0.56**	−0.32**	0.66**	−0.34**	(0.76)					
(24) Supervisor support HA	0.24**	0.26**	0.28**	0.12**	0.38**	0.06	−0.34**	0.72**	−0.35**	0.76**	−0.46**	(0.89)				
(25) Feedback CA	0.14**	0.02	0.04	0.04	–0.04	0.21**	0.51**	−0.23**	0.57**	−0.25**	0.56**	−0.26**	(0.77)			
(26) Feedback HA	0.22**	0.30**	0.29**	0.15**	0.39**	0.06	−0.355**	0.77**	−0.24**	0.72**	−0.29**	0.72**	−0.33**	(0.91)		
(27) Burnout	–0.03	0.20**	–0.04	0.23**	–0.03	0.13**	−0.17**	0.19**	−0.18**	0.24**	−0.20**	0.23**	−0.23**	0.24**	(0.92)	
(28) Engagement	0.25**	–0.04	0.24**	−0.12**	0.27**	–0.08	0.13**	0.17**	0.20**	0.06	0.21**	0.08	0.25**	0.12**	−0.60**	(0.93)

CA, challenge appraisal; HA, hindrance appraisal. Reliability estimates (alpha) between brackets on the diagonal.*p < 0.05; **p < 0.01 level (two-tailed).

#### Hypotheses Testing

##### Appraisals of Job Demands and Well-Being

We hypothesized that job demands are positively associated with burnout and negatively associated with engagement (Hypothesis 1). Table 3 shows that time urgency (β = 0.42, *p* < 0.001), role conflict (β = 0.45, *p* < 0.001), and emotional demands (β = 0.59, *p* < 0.001) were positively related to burnout. In contrast, time urgency (β = –0.20, *p* < 0.001), role conflict (β = –0.23, *p* < 0.001), and emotional demands (β = –0.32, *p* < 0.001) were negatively associated with engagement. Hence, Hypothesis 1 was supported.

**TABLE 3:** Regression results for the moderation of appraisals on the relationships between job demands and work engagement/Burnout in Study 1.

	**Step 1**		**Step 2**		**Step 3**	
**Predictors**	**Burnout**	**Engagement**	**Burnout**	**Engagement**	**Burnout**	**Engagement**
Work Time	0.05	−0.20***	–0.01	−0.13**	–0.00	−0.13**
Education	–0.00	0.13**	–0.07	0.13**	–0.07	0.13**
*Time Urgency*			0.42***	−0.20***	0.42***	−0.20***
Challenge Appraisals			–0.02	0.29***	–0.04	0.31***
Hindrance Appraisals			0.14**	0.10*	0.15**	0.09
Time Urgency × CA					–0.07	0.10*
Time Urgency × HA					0.10*	–0.07
*R* ^2^	0.00	0.06	0.20	0.14	0.23	0.16
Work Time	0.05	−0.20***	0.03	−0.16***	0.03	−0.15***
Education	–0.00	0.13**	–0.04	0.13**	–0.05	0.13**
*Role Conflict*			0.45***	−0.23***	0.45***	−0.22***
Challenge Appraisals			–0.04	0.26***	–0.05	0.27***
Hindrance Appraisals			0.18***	0.01	0.19	–0.00
Role Conflict × CA					–0.03	0.13**
Role Conflict × HA					–0.05	0.02
*R* ^2^	0.002	0.06	0.25	0.15	0.25	0.16
Work Time	0.05	−0.20***	0.01	–0.13	0.00	−0.12***
Education	0.00	0.13**	–0.02	0.11**	–0.01	0.11**
*Emotional Demands*			0.59***	−0.32***	0.59***	−0.32***
Challenge Appraisals			–0.03	0.32***	–0.04	0.33***
Hindrance Appraisals			0.03	0.13**	0.05	0.13**
Emotional Demands × CA					0.01	0.11*
Emotional Demands × HA					0.12**	–0.01
*R* ^2^	0.00	0.06	0.35	0.21	0.37	0.23

*p < 0.05, **p < 0.01, ***p < 0.001. CA, challenge appraisal; HA, hindrance appraisal. Standardized regression coefficients were reported.

**FIGURE 1:**
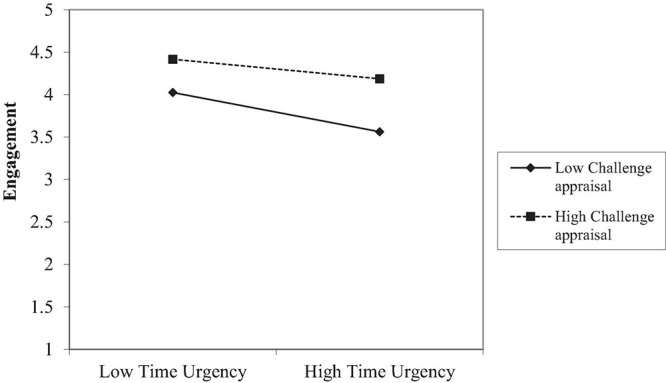
The interaction between time urgency and challenge appraisal on engagement in Study 1.

Then, we tested the moderating effects of appraisals on the relationship between various job demands and work engagement/burnout (Hypotheses 2–5). The interactions between challenge appraisals and time urgency (β = 0.10, *p* < 0.05), role conflict (β = 0.13, *p* < 0.05), and emotional demands (β = 0.11, *p* < 0.05), significantly predict work engagement. Follow-up tests showed that the adverse effects of job demands on engagement were weaker when challenge appraisals of job demands were high (time urgency, *b* = –0.09, *p* = 0.08; role conflict, *b* = –0.09, *p* = 0.07; emotional demands, *b* = –0.33, *p* < 0.001) than when these appraisals were low (time urgency, *b* = –0.25, *p* < 0.001; role conflict, *b* = –0.26, *p* < 0.001; emotional demands, *b* = –0.63, *p* < 0.001). We plotted the simple slope analysis for time urgency in Figure 1 (the figures for role conflict and emotional demands analyses are presented in the Supplementary File; the patterns are similar to those in Figure 1). Hence, Hypothesis 2 was supported.

**FIGURE 2:**
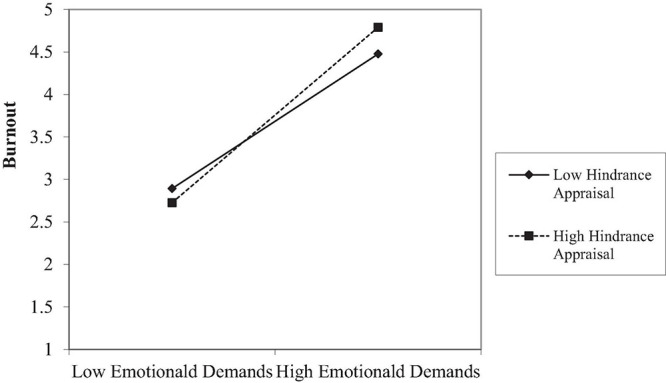
The interaction between emotional demands and hindrance appraisal on burnout in Study 1.

Contrary to our expectations, no significant moderation effects of challenge appraisal and job demands on burnout were found (Hypothesis 3 not supported). Similarly, the interaction effects between hindrance appraisal and job demands on engagement were not significant (Hypothesis 4 not supported). In addition, while the interaction between hindrance appraisal and role conflict failed to predict burnout (β = –0.05, *p* = 0.27), the interactions of hindrance appraisal and time urgency (β = 0.10, *p* < 0.05) and emotional demands (β = 0.12, *p* < 0.05) predicted burnout. As expected, the simple slope tests showed that the regression coefficients of job demands on burnout were stronger when hindrance appraisal was high (time urgency, *b* = 0.45, *p* < 0.001; emotional demands, *b* = 1.08, *p* < 0.001) than when hindrance appraisal was low (time urgency, *b* = 0.29, *p* < 0.001; emotional demands, *b* = 0.73, *p* < 0.001; cf. Figure 2. For brevity, we only present the plot for emotional demands, the plot of time urgency, which is similar to Figure 2, is presented in the Supplementary File). Hence, Hypothesis 5 was partially supported.

##### Appraisals of Job Resources and Well-Being

Hypothesis 6 stated that job resources will be positively associated with work engagement and negatively to burnout. As shown in Table 4, engagement was positively related to autonomy (β = 0.34, *p* < 0.001), supervisor support (β = 0.42, *p* < 0.001), colleague support (β = 0.33, *p* < 0.001), and feedback from others (β = 0.31, *p* < 0.001). Burnout was negatively associated with autonomy (β = –0.36, *p* < 0.001), supervisor support (β = –0.41, *p* < 0.001), colleague support (β = –0.30, *p* < 0.001), and feedback from others (β = –0.28, *p* < 0.001). These results support Hypothesis 6.

**TABLE 4:** Regression results for the moderation of appraisals on the relationships between job resources and work engagement/Burnout in Study 1.

	**Step 1**		**Step 2**		**Step 3**	
**Predictors**	**Burnout**	**Engagement**	**Burnout**	**Engagement**	**Burnout**	**Engagement**
Work Time	0.05	−0.20***	0.07	−0.13**	0.06	−0.13**
Education	0.00	0.13***	0.03	0.07	0.03	0.07
*Autonomy*			−0.36***	0.34***	−0.31***	0.30***
Challenge Appraisals			–0.07	0.23***	–0.07	0.22***
Hindrance Appraisals			0.20***	0.21***	0.13*	0.28***
Autonomy × CA					–0.05	0.06
Autonomy × HA					0.12*	–0.10
*R* ^2^	0.00	0.06	0.18	0.24	0.19	0.25
Work Time	0.05	−0.20***	0.03	−0.12**	0.04	−0.12**
Education	0.00	0.13**	0.02	0.09*	0.02	0.09*
*Supervisor Support*			−0.41***	0.42***	−0.37***	0.40***
Challenge Appraisals			–0.01	0.18***	–0.03	0.18***
Hindrance Appraisals			0.20***	0.17***	0.18***	0.19***
Supervisor Support × CA					–0.06	–0.01
Supervisor Support × HA					0.15**	−0.15**
*R* ^2^	0.00	0.06	0.22	0.29	0.26	0.31
Work Time	0.05	−0.20***	0.07	−0.16***	0.07	−0.16***
Education	–0.00	0.13**	0.01	0.11**	0.00	0.11**
*Colleague Support*			−0.30**	0.33***	−0.28***	0.31***
Challenge Appraisals			–0.04	0.16***	–0.04	0.16***
Hindrance Appraisals			0.21**	0.13**	0.21***	0.13**
Colleague Support × CA					–0.01	0.04
Colleague Support × HA					0.15**	−0.12**
*R* ^2^	0.00	0.06	0.16	0.21	0.19	0.23
Work Time	0.05	−0.20***	0.09*	−0.17***	0.10*	−0.18***
Education	0.00	0.13**	0.02	0.08*	0.02	0.08*
*Feedback*			−0.28***	0.31***	−0.24***	0.28***
Challenge Appraisals			−0.10*	0.24***	−0.12**	0.25***
Hindrance Appraisals			0.19***	0.20***	0.18***	0.21***
Feedback × CA					–0.08	0.02
Feedback × HA					0.16***	−0.11**
*R* ^2^	0.00	0.06	0.17	0.24	0.20	0.25

*p < 0.05,**p < 0.01, ***p < 0.001. CA, challenge appraisal; HA, hindrance appraisal. Standardized regression coefficients were reported.

**FIGURE 3:**
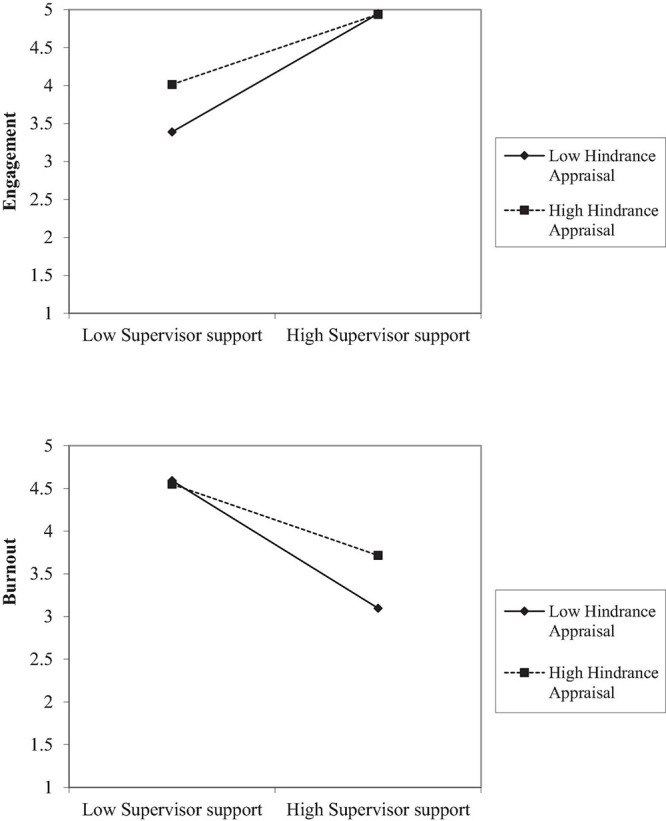
The interactions between supervisor support and hindrance appraisal on engagement **(top)** and burnout **(bottom)** in Study 1.

Next, we tested the moderating effects of appraisals on the relationship between job resources and work engagement/burnout (Hypotheses 7–10). Unexpectedly, no significant moderation effects of *challenge* appraisals and job resources on burnout and engagement were found (Hypotheses 7–8 not supported). Conversely, the interactions of *hindrance* appraisals and autonomy (β = –0.10, *p* = 0.06), supervisor support (β = –0.15, *p* < 0.001), colleague support (β = –0.12, *p* < 0.05), and feedback from others (β = –0.11, *p* < 0.05) predicted work engagement. Follow-up simple slope tests (Figure 3) showed that when hindrance appraisal was high (autonomy, *b* = 0.16, *p* < 0.01; supervisor support, *b* = 0.41, *p* < 0.001; colleague support, *b* = 0.31, *p* < 0.01; feedback from others, *b* = 0.26, *p* < 0.01), the positive relations between engagement and these resources were weaker than when hindrance appraisal was low (autonomy, *b* = 0.31, *p* < 0.001; supervisor support, *b* = 0.84, *p* < 0.001; colleague support, *b* = 0.70, *p* < 0.001; feedback from others, *b* = 0.58, *t* = 7.34, *p* < 0.001). Thus, Hypothesis 9 was supported.

Lastly, the interactions of hindrance appraisals and autonomy (β = 0.12, *p* < 0.05), supervisor support (β = 0.15, *p* < 0.001), colleague support (β = 0.15, *p* < 0.001), and feedback from others (β = 0.16, *p* < 0.001) predicted burnout, such that when hindrance appraisal was high, the negative effect of job resources on burnout was weaker. Follow-up simple slope tests showed that the regression coefficients of job resources on burnout were weaker when hindrance appraisal was high (autonomy, *b* = –0.17, *p* < 0.05; supervisor support, *b* = –0.35, *p* < 0.001; colleague support, *b* = –0.22, *p* < 0.05; feedback from others, *b* = –0.14, *p* = 0.15) than when hindrance appraisal was low (autonomy, *b* = –0.34, *p* < 0.001; supervisor support, *b* = –0.81, *p* < 0.001; colleague support, *b* = –0.72, *p* < 0.001; feedback from others, *b* = –0.60, *p* < 0.001; see Figure 3. We only plotted the simple slope analysis results for supervisor support; the other moderation patterns are similar to Figure 3 and are plotted in the Supplementary File). Therefore, Hypothesis 10 was supported.

### Summary of Study 1 Findings

The results of Study 1 reveal that job characteristics that are usually categorized as “demands” (i.e., time urgency, role conflict, and emotional demands) or “resources” (i.e., autonomy, social support from supervisors and colleagues, and feedback from others) can be appraised as both challenging and hindering. Further, the moderation analysis showed 12 significant interaction effects between job characteristics and appraisals. Specifically, our results indicate that individuals appraisals of job characteristics matter: a positive interpretation (challenge appraisal) of job demands will buffer its detrimental effect on work engagement such that when challenge appraisal was high, the negative relationship between job demands and engagement became weaker; whereas a negative interpretation of job demands (time urgency and emotional demands) will strengthen its detrimental effects on burnout. Conversely, a negative interpretation (hindrance appraisal) of job resources will undermine its beneficial effects such that when hindrance appraisal was high, the positive/negative relationship between job resources and work engagement/burnout became weaker.

The study provided preliminary support for our hypotheses. However, there are several limitations to Study 1. First, we measured employees’ appraisal in scenarios, which might be inferior to assessing their appraisals of actual job characteristics. Second, we collected data from a multi-occupation sample, which implies that there may have been subtle differences in the job characteristics of the participants. For example, for technology employees, the meaning of emotional demands may be different from for nurses (Bakker and Sanz-Vergel, 2013). Third, we collected data using an online panel. Although there are some important advantages to such an approach (Porter et al., 2018), participants’ experiences of participating in many different surveys might have impacted their answers due to a practice effect (i.e., an improvement in performance on a task due to repetition) or a fatigue effect (i.e., a decrease in performance of a task due to boredom or tiredness; Wesnes and Pincock, 2002). Finally, because of the cross-sectional nature of the data (Podsakoff et al., 2003), we were unable to make causal conclusions about the relationships among the variables.

## Study 2 Method

To address the limitations of Study 1, we collected data from a group of nurses working in a single hospital in China to provide an additional test of the hypotheses stated in Study 1. By doing so, we aim to increase the generalizability of our findings since this is a homogenous rather than a heterogeneous sample from multiple organizations. This follow-up study used a different approach for measuring appraisals (i.e., referring to employees’ current job characteristics instead of referring to a scenario). In this vein, Study 2 aims to both cross-validate and extend the findings obtained in Study 1.

### Sample and Procedure

We collected data from different departments within a Chinese hospital. We sent 400 online questionnaires, 316 of which were returned (a response rate of 79%). Participants were predominantly female (61.4%), and were on average 31.4 years old. They had been employed in their current organization for on average 6.33 years. Informed consent was obtained and participants were ensured anonymity. As a reward, participants received 15 RMB (about €2) for their participation.

### Measures

We measured *time urgency*, *emotional demands*, *autonomy*, *colleague support*, *work engagement*, and *burnout* with the same items as in Study 1. With regards to *appraisal*, we instructed participants to appraise their own job characteristics. As an example, when measuring emotional demands, we asked “Think about the amount of emotional demands you are experiencing in the last 2 weeks in your work. Could you please indicate how you would consider the emotional demands in your job? I believe that the emotional demands in my job…” For the measurement of challenge and hindrance appraisals, we used the same eight items as in Study 1. The 2-week time frame was used as researchers suggested that appraisals should be framed in related to an event (and/or a time frame in which events may occur) so respondents can understand what they are appraising (Searle and Auton, 2015), and this time frame was previously used in Milner et al. (2017). Table 5 shows the Cronbach’s alphas of these scales (ranged from 0.71 to 0.94), demonstrating adequate reliability.

**TABLE 5:** Means, standard deviations, and correlations among the study variables in Study 2.

**Variable**	**Mean**	***SD***	**Skewness**	**Kurtosis**	**1**	**2**	**3**	**4**	**5**	**6**	**7**	**8**	**9**	**10**	**11**	**12**
(1) Age	31.40	7.27	1.36	2.18	1											
(2) Gender	1.61	0.49	–0.47	–1.79	−.19**	1										
(3) Education	3.98	0.59	–0.66	2.77	−0.15**	0.05	1									
(4) Work time	42.18	15.28	–0.51	1.78	–0.03	–0.11	0.04	1								
(5) Tenure	6.33	5.32	1.69	3.15	0.77**	–0.10	−0.20**	–0.10	1							
(6) Time urgency	4.27	1.25	–0.35	–0.59	–0.02	0.00	0.09	0.02	–0.06	(0.91)						
(7) Role conflict	4.16	1.37	–0.22	–0.80	−0.13*	–0.07	0.02	–0.02	−0.14*	0.49**	(0.86)					
(8) Emotional demand	2.80	0.94	0.11	–0.68	–0.09	0.14*	0.03	0.01	–0.10	0.54**	0.48**	(0.82)				
(9) Autonomy	4.45	1.40	–0.27	–0.81	0.16**	0.03	–0.04	−0.11*	0.14*	−0.32**	−0.41**	−0.26**	(0.92)			
(10) Colleague support	3.47	0.71	–0.55	0.30	0.10	–0.02	–0.01	–0.05	0.11*	−0.28**	−0.21**	−0.29**	0.39**	(0.71)		
(11) Supervisor support	3.20	0.84	–0.29	–0.40	0.15**	–0.03	–0.02	−0.13*	0.16**	−0.24**	−0.26**	−0.25**	0.44**	0.68**	(0.78)	
(12) Feedback	3.54	0.87	–0.82	0.44	0.11	–0.03	0.09	–0.08	0.09	–0.08	−0.14*	−0.13*	0.20**	0.45**	0.53**	(0.79)
(13) Time urgency CA	5.07	1.05	–0.85	0.85	0.13*	–0.02	0.04	−0.13*	0.11	−0.13*	−0.19**	−0.27**	0.42**	0.51**	0.50**	0.36**
(14) Time urgency HA	3.68	1.16	0.14	–0.24	–0.03	0.03	0.08	0.04	–0.05	0.40**	0.40**	0.46**	−0.35**	−0.39**	−0.37**	−0.21**
(15) Role conflict CA	4.57	1.27	–0.47	–0.23	0.15**	0.09	–0.02	−0.12*	0.11*	0.00	–0.03	–0.03	0.30**	0.38**	0.41**	0.31**
(16) Role conflict HA	4.11	1.35	–0.24	–0.57	–0.06	–0.02	0.09	0.03	–0.05	0.22**	0.24**	0.27**	−0.24**	−0.28**	−0.32**	−0.18**
(17) Emotional demand CA	4.63	1.30	–0.49	–0.29	0.12*	0.03	0.00	−0.11*	0.08	–0.06	–0.09	−0.14*	0.34**	0.43**	0.49**	0.30**
(18) Emotional demand HA	3.91	1.40	0.01	–0.86	–0.01	0.00	0.12*	0.01	–0.04	0.27**	0.27**	0.29**	−0.21**	−0.20**	−0.26**	−0.13*
(19) Autonomy CA	5.50	0.83	–0.88	2.03	0.02	0.00	–0.01	−0.11*	0.05	−0.11*	−0.15**	–0.09	0.27**	0.30**	0.26**	0.13*
(20) Autonomy HA	2.96	1.25	0.69	0.09	0.02	0.01	0.06	–0.09	–0.02	0.30**	0.38**	0.27**	−0.17**	−0.11*	–0.06	–0.01
(21) Colleague support CA	5.37	0.86	–0.65	2.29	–0.03	0.02	–0.03	–0.05	–0.02	−0.15**	−0.14*	−0.18**	0.23**	0.38**	0.30**	0.19**
(22) Colleague support HA	2.88	1.19	0.80	0.54	0.08	–0.04	0.03	–0.07	0.03	0.20**	0.22**	0.18**	–0.04	–0.02	0.08	0.02
(23) Supervisor support CA	5.43	0.90	–0.99	2.64	–0.01	–0.04	0.07	–0.10	–0.02	−0.13*	−0.12*	−0.21**	0.28**	0.32**	0.35**	0.25**
(24) Supervisor support HA	2.85	1.21	0.92	0.58	0.08	0.02	0.02	–0.09	0.00	0.29**	0.25**	0.23**	–0.06	–0.06	–0.05	–0.01
(25) Feedback CA	5.46	0.83	–0.90	3.10	–0.04	0.00	0.00	0.00	0.00	–0.08	−0.13*	–0.09	0.26**	0.30**	0.24**	0.20**
(26) Feedback HA	2.80	1.17	0.88	0.74	0.10	0.00	0.05	−0.12*	0.03	0.26**	0.27**	0.20**	−0.11*	−0.11*	–0.02	0.02
(27) Burnout	2.50	1.15	0.35	0.02	−0.16**	0.08	0.02	0.02	−0.15**	0.49**	0.48**	0.62**	−0.42**	−0.44**	−0.44**	−0.30**
(28) Engagement	3.20	1.12	0.05	–0.46	0.22**	–0.05	0.04	0.00	0.17**	−0.27**	−0.27**	−0.43**	0.43**	0.50**	0.49**	0.38**

**Variable**	**13**	**14**	**15**	**16**	**17**	**18**	**19**	**20**	**21**	**22**	**23**	**24**	**25**	**26**	**27**	**28**

(13) Time urgency CA	(0.85)															
(14) Time urgency HA	−0.55**	(0.85)														
(15) Role conflict CA	0.62**	−0.28**	(0.89)													
(16) Role conflict HA	−0.37**	0.57**	−0.58**	(0.91)												
(17) Emotional demand CA	0.68**	−0.42**	0.72**	−0.47**	(0.90)											
(18) Emotional demand HA	−0.28**	0.64**	−0.31**	0.71**	−0.53**	(0.92)										
(19) Autonomy CA	0.45**	−0.17**	0.25**	–0.04	0.30**	–0.03	(0.82)									
(20) Autonomy HA	–0.08	0.38**	0.09	0.28**	0.02	0.32**	−0.40**	(0.93)								
(21) Colleague support CA	0.49**	−0.25**	0.35**	−0.20**	0.38**	–0.09	0.62**	−0.25**	(0.83)							
(22) Colleague support HA	–0.04	0.31**	0.12*	0.28**	0.12*	0.31**	−0.29**	0.62**	−0.25**	(0.91)						
(23) Supervisor support CA	0.54**	−0.32**	0.35**	−0.22**	0.36**	−0.16**	0.58**	−0.25**	0.72**	−0.27**	(0.86)					
(24) Supervisor support HA	–0.09	0.33**	0.11	0.25**	0.10	0.30**	−0.27**	0.67**	−0.27**	0.74**	−0.42**	(0.92)				
(25) Feedback CA	0.48**	−0.25**	0.37**	−0.17**	0.37**	–0.10	0.61**	−0.26**	0.71**	−0.21**	0.65**	−0.23**	(0.83)			
(26) Feedback HA	–0.10	0.34**	0.07	0.26**	0.03	0.28**	−0.35**	0.65**	−0.34**	0.71**	−0.30**	0.73**	−0.44**	(0.92)		
(27) Burnout	−0.42**	0.53**	−0.24**	0.36**	−0.34**	0.38**	−0.27**	0.29**	−0.36**	0.21**	−0.35**	0.29**	−0.33**	0.30**	(0.94)	
(28) Engagement	0.56**	−0.51**	0.34**	−0.30**	0.47**	−0.31**	0.32**	−0.18**	0.41**	–0.09	0.41**	−0.16**	0.37**	−0.15**	−0.75**	(0.94)

CA, challenge appraisal; HA, hindrance appraisal. Reliability estimates (alpha) between brackets on the diagonal.*p < 0.05; **p < 0.01 level (two-tailed).

### Study 2 Results

#### Measurement Model

Table 6 shows that fit indices of the hypothesized 23-factor model had reasonable fit indexes [χ^2^_(3232)_ = 6,288.12, *p* < 0.001; RMSEA = 0.05; CFI = 0.87; TLI = 0.85; SRMR = 0.05] and fit the data better than five alternative models (see Table 6). In addition, we tested a model where an additional unmeasured latent method factor was included. The results showed that the common method factor explained 5.3% of the variance, indicating that the method effects were not severe.

**TABLE 6:** Results of confirmatory factor analyses in Study 2.

**Model**	**Chi-Square**	***df***	**RMSEA [90% CI]**	**CFI**	**TLI**	**SRMR**	**AIC**	**BIC**
23-factor parceled	6288.12	3316	0.05 [0.051,0.055]	0.87	0.85	0.05	70840.61	72759.80
23-factor not parceled	8297.52	4499	0.05 [0.05,0.053]	0.86	0.84	0.05	82427.79	84493.45
26 factor	8134.40	4427	0.05 [0.05,0.053]	0.86	0.85	0.05	82408.66	84744.73
11-factor	12367.98	3514	0.09 [0.088,0.091]	0.60	0.59	0.12	76524.48	77700.02
13-factor	9051.57	3491	0.07 [0.069,0.073]	0.75	0.74	0.07	73254.06	74516.00
8-factor	10455.34	3541	0.08 [0.077,0.80]	0.69	0.68	0.08	74557.84	75631.98
23-factor parceled with common method effects	6268.23	3315	0.05 [0.051,0.055]	0.87	0.85	0.05	70822.73	72745.67
1-factor	19441.43	3569	0.12 [0.117,0.120]	0.29	0.27	0.15	83487.93	84456.91

*RMSEA, root mean square error of approximation; CFI, comparative fit index; TLI, Tucker–Lewis index; SRMR, standardized root mean residual; CI, confidence interval; AIC, Akaike information criterion; BIC, Bayesian information criterion.*

*23-factor model-parceled: three demands: time urgency, role conflict, and emotional demands; four resources: autonomy, feedback, colleague support, and supervisor support; seven challenge and seven hindrance appraisals of demands and resources: and two outcomes with nine engagement items were mean-parceled as three indicators based on the three engagement dimensions and loading on one latent engagement factor; nine burnout items were mean-parceled as two indicators representing exhaustion and cynicism, and loading on one latent burnout factor);*

*23-factor not parceled: 23-factor model-parceled with nine items of engagement loaded on one, and nine items of burnout loaded on another latent factor.*

*26-factor: 23-factor not parceled with two outcomes loaded as five factors: vigor, dedication, absorption, exhaustion, and cynicism.*

*11-factor: 23-factor model-parceled with seven challenge appraisals into one, and seven hindrance appraisals loaded on one factor.*

*13-factor: 11-factor splitting appraisals as four factors: challenge/hindrance appraisals of demands and challenge/hindrance appraisals of resources.*

*8-factor: 11-factor combining three demands into one factor, and four resources into another factor.*

*23-factor parceled with common method effects: 23-factor model-parceled added a latent method factor allowing all items loaded on the method factor.*

*1-factor model: with all variables loaded onto one factor.*

#### Challenge and Hindrance Ratings of Job Characteristics

Table 5 presents the means, standard deviations, internal consistency reliabilities, and zero-order correlations for the manifest scale scores. This table shows that time urgency was considered to be more of a challenge (*M* = 5.07, *SD* = 1.05) than of a hindrance (*M* = 3.68, *SD* = 1.16; *T* = 12.73, *p* < 0.001). Similarly, role conflict (*M*_challenge_ = 4.57, *SD* = 1.27; *M*_hindrance_ = 4.11, *SD* = 1.35; *T* = 3.50, *p* < 0.001) and emotional demands (*M*_challenge_ = 4.63, *SD* = 1.30; *M*_hindrance_ = 3.91, *SD* = 1.40; *T* = 5.47, *p* < 0.001) were more often perceived as challenges than as hindrances. As for job resources, the results in Table 5 show that autonomy was considered more as a challenge (*M* = 5.50, *SD* = 0.83) than as a hindrance (*M* = 2.96, *SD* = 1.25; *T* = 25.68, *p* < 0.001). Similar results were found for supervisor support (*M*_challenge_ = 5.43, *SD* = 0.90; *M*_hindrance_ = 2.85, *SD* = 1.21; *T* = 25.72, *p* < 0.001), feedback from others (*M*_challenge_ = 5.46, *SD* = 0.83; *M*_hindrance_ = 2.80, *SD* = 1.17; *T* = 27.76, *p* < 0.001), and colleague support (*M*_challenge_ = 5.37, *SD* = 0.86; *M*_hindrance_ = 2.88, *SD* = 1.19; *T* = 27.11, *p* < 0.001). Since the *SD*s for these appraisal ratings do not equal zero, this demonstrates that employees may differ quite strongly in their appraisals of these job characteristics. These findings also show that job characteristics can be both appraised as challenges and hindrances, but in general more as a challenge than as a hindrance (see Table 5).

#### Hypotheses Testing

Hypothesis 1 postulated that job demands are positively associated with burnout and negatively associated with engagement. The results show that time urgency (β = 0.35, *p* < 0.001), role conflict (β = 0.43, *p* < 0.001), and emotional demands (β = 0.56, *p* < 0.001) were positively related to burnout. In contrast, time urgency (β = –0.13, *p* < 0.01), role conflict (β = –0.24, *p* < 0.001), and emotional demands (β = –0.38, *p* < 0.001) were negatively associated with engagement, which again supporting Hypothesis 1.

**FIGURE 4:**
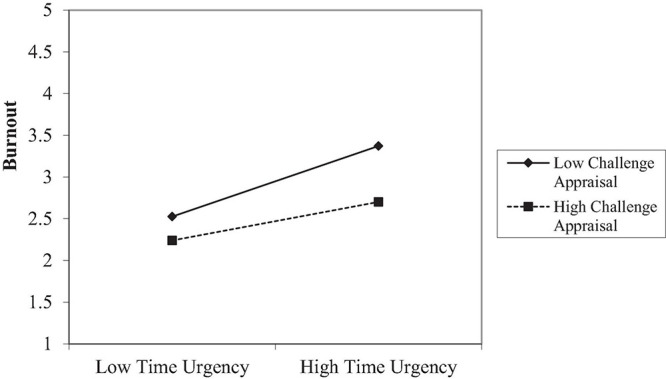
The interaction between time urgency and challenge appraisal on burnout in Study 2.

Then, we tested the interaction effects between various job demands and appraisals on work engagement/burnout (Hypotheses 2–5). The results in Table 7 show that the interactions between challenge appraisals and time urgency (β = –0.13, *p* < 0.05), role conflict (β = –0.102, *p* = 0.08), and emotional demands (β = –0.12, *p <* 0.05) significantly predicted burnout. We plotted the simple slopes for time urgency in Figure 4 (the interaction pattern for emotional demands was similar to time urgency and the interaction figure is provided in the Supplementary Materials). The detrimental effect of time urgency on burnout was weaker when challenge appraisal was high, which partially supported Hypothesis 3. No other interactions were found between job demands and appraisals on the outcomes. Hence, Hypotheses 2, 4, and 5 were not supported.

**TABLE 7:** Regression results for the moderation of appraisals on the relationships between job demands and work engagement/burnout in Study 2.

	**Step 1**		**Step 2**		**Step3**	
**Predictors**	**Burnout**	**Engagement**	**Burnout**	**Engagement**	**Burnout**	**Engagement**
Work Time	0.02	–0.00	–0.03	0.06	–0.03	0.064
Education	0.020	0.04	–0.02	0.06	–0.02	0.06
*Time Urgency*			0.35***	−0.13**	0.35***	−0.12*
Challenge Appraisals			−0.23***	0.42***	−0.22***	0.41***
Hindrance Appraisals			0.26***	−0.24***	0.27***	−0.25***
Time Urgency × CA					−0.13*	0.04
Time Urgency × HA					–0.04	0.08
*R* ^2^	0.00	0.00	0.41	0.40	0.42	0.40
Work Time	0.02	–0.00	0.01	0.03	0.01	0.03
Education	0.02	0.04	–0.01	0.06	–0.00	0.05
*Role Conflict*			0.43***	−0.24***	0.43***	−0.24***
Challenge Appraisals			–0.11	0.30***	–0.10	0.29***
Hindrance Appraisals			0.19**	–0.08	0.21**	–0.09
Role Conflict × CA					–0.10	0.09
Role Conflict × HA					–0.00	–0.01
*R* ^2^	0.00	0.00	0.30	0.19	0.31	0.20
Work Time	0.02	–0.00	–0.01	0.05	–0.01	0.05
Education	0.02	0.04	–0.01	0.05	–0.01	0.04
*Emotional Demands*			0.56***	−0.38***	0.54***	−0.36***
Challenge Appraisals			−0.21***	0.44***	−0.19***	0.42***
Hindrance Appraisals			0.11*	0.02	0.13*	0.01***
Emotional Demands × CA					−0.12*	0.10
Emotional Demands × HA					–0.04	0.02
*R* ^2^	0.00	0.00	0.46	0.36	0.47	0.37

*p < 0.05, **p < 0.01, ***p < 0.001. CA, challenge appraisal; HA, hindrance appraisal. Standardized regression coefficients were reported.

Hypothesis 6 stated that job resources will be positively associated with work engagement and negatively with burnout. Table 8 shows that autonomy (β = 0.38, *p* < 0.001), supervisor support (β = 0.40, *p* < 0.001), colleague support (β = 0.41, *p* < 0.001), and feedback from others (β = 0.32, *p* < 0.001) was positively related to engagement. Autonomy (β = –0.36, *p* < 0.001), supervisor support (β = –0.38, *p* < 0.001), colleague support (β = –0.36, *p* < 0.001), and feedback from others (β = –0.27, *p* < 0.001) were negatively associated with burnout. These results supported Hypothesis 6.

**TABLE 8:** Regression results for the moderation of appraisals on the relationships between job resources and work engagement/burnout in Study 2.

	**Step 1**		**Step 2**		**Step 3**	
**Predictors**	**Burnout**	**Engagement**	**Burnout**	**Engagement**	**Burnout**	**Engagement**
Work Time	0.02	–0.00	–0.01	0.06	–0.01	0.06
Education	0.02	0.04	–0.01	0.06	–0.01	0.06
*Autonomy*			−0.36***	0.38***	−0.36***	0.36***
Challenge Appraisals			–0.10	0.21***	–0.09	0.23***
Hindrance Appraisals			0.19**	–0.03	0.19**	–0.04
Autonomy × CA					0.02	0.13*
Autonomy × HA					–0.00	0.07
*R* ^2^	0.00	0.00	0.23	0.24	0.23	0.25
Work Time	0.02	–0.00	–0.02	0.07	–0.02	0.07
Education	0.02	0.04	0.02	0.03	0.01	0.06
*Supervisor Support*			−0.38***	0.40***	−0.37***	0.39***
Challenge Appraisals			−0.14*	0.26***	−0.15*	0.29***
Hindrance Appraisals			0.21***	–0.02	0.21***	–0.03
Supervisor Support × CA					–0.05	0.15**
Supervisor Support × HA					0.02	0.04
*R* ^2^	0.00	0.00	0.28	0.31	0.28	0.33
Work Time	0.02	–0.00	0.00	0.03	–0.01	0.04
Education	0.02	0.04	0.01	0.05	0.00	0.06
*Colleague Support*			−0.36***	0.41***	−0.36***	0.39***
Challenge Appraisals			−0.19**	0.26***	−0.20***	0.29***
Hindrance Appraisals			0.15**	–0.01	0.14**	–0.01
Colleague Support × CA					–0.03	0.11*
Colleague Support × HA					0.03	0.01
*R* ^2^	0.00	0.00	0.26	0.31	0.26	0.32
Work Time	0.02	–0.00	0.02	0.02	0.01	0.03
Education	0.02	0.04	0.03	0.02	0.01	0.03
*Feedback*			−0.27***	0.32***	−0.26***	0.31***
Challenge Appraisals			−0.17**	0.30***	−0.13*	0.26***
Hindrance Appraisals			0.23***	–0.03	0.25***	–0.06
Feedback × CA					−0.22***	0.19**
Feedback × HA					–0.04	0.09
*R* ^2^	0.00	0.00	0.21	0.24	0.25	0.26

*p < 0.05, **p < 0.01, ***p < 0.001. CA, challenge appraisal; HA, hindrance appraisal. Standardized regression coefficients were reported.

**FIGURE 5:**
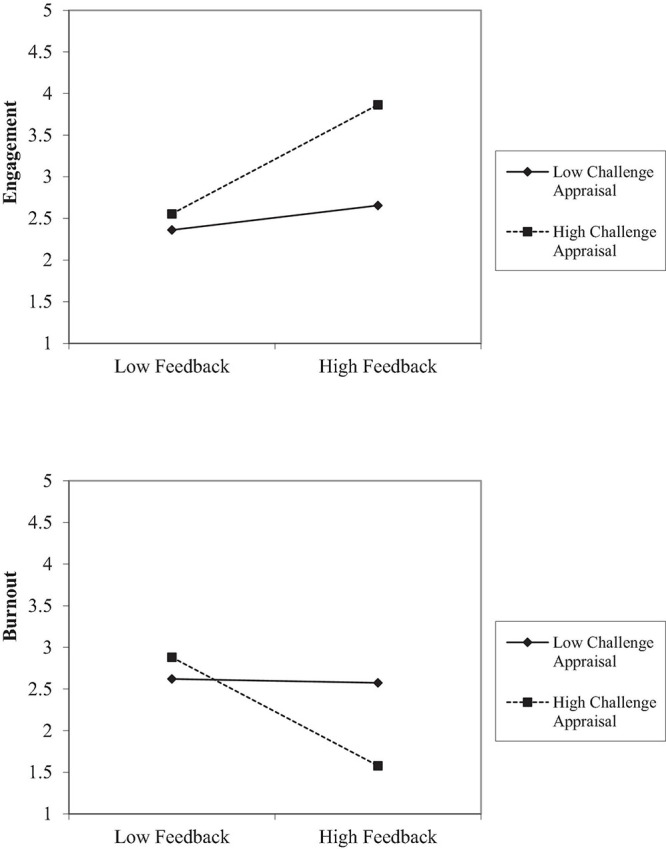
The interactions between feedback and challenge appraisal on engagement **(top)** and burnout **(bottom)** in Study 2.

Finally, we tested the moderating effects of appraisals on the relationship between various job resources and work engagement/burnout (Hypotheses 7–10). The results show that the interactions between challenge appraisal and autonomy (β = 0.13, *p <* 0.05), supervisor support (β = 0.15, *p <* 0.01), colleague support (β = 0.11, *p <* 0.05), and feedback (β = 0.19, *p <* 0.01) significantly predicted employee engagement. Specifically, the positive relationship between these job characteristics and work engagement was more positive when challenge appraisal was high (see Figure 5 for feedback; other interaction effects are similar to feedback and are presented in the Supplementary File), which supported Hypothesis 7. The interactions between challenge appraisal and job resources on burnout was significant only for feedback (β = –0.22, *p <* 0.001). The negative relationship between feedback and burnout was stronger when challenge appraisal was high (see Figure 5). Therefore, Hypothesis 8 was partially supported. Unexpectedly, no significant moderation effects of *hindrance* appraisals and job resources on burnout and engagement were found (Hypotheses 9–10 not supported).

#### Control Variables

We also tested our hypotheses by including some control variables (i.e., age, gender, education, tenure, and work time) and the pattern of results did not change, supporting the robustness of the findings.

### Summary of Study 2 Findings

In Study 2, we used a homogeneous sample to replicate our findings in Study 1. The results in Study 2 supported our argument that job characteristics can be appraised simultaneously as challenges and hindrances, and that such appraisals moderate some of the job characteristics – employee well-being relationships. The moderation results showed that a positive interpretation of job demands (time urgency and emotional demands) mitigates its detrimental effect on burnout. In particular, when challenge appraisal of job demands is high, the negative relationship between job demands and burnout became weaker (partially supported Hypothesis 3). In addition, a positive interpretation of job resources (autonomy, supervisor support, colleague support, and feedback) will strengthen its benefit on employee work engagement and burnout (only for feedback). When challenge appraisal of job resources is high, the positive/negative relationship between job resources and work engagement/burnout became stronger (sully supported Hypothesis 7 and partially supported Hypothesis 8). However, other hypothesized interaction effects between job characteristics and appraisals on employee well-being were not supported by our data (Hypotheses 2, 4, 5, 9, and 10 were not supported). Therefore, the moderation hypotheses were partially supported across the two studies with different samples and study designs (i.e., measurement of appraisals), and the significant relationships across the two studies are in line with the directions of the links predicted in our hypotheses. Note that in Study 2, the sample sizes were relatively small compared to Study 1, and the model fit indices of CFI and TLI for CFA were lower than suggested (Hu and Bentler, 1999). This should be considered a limitation.

## Overall Discussion

This study focused on the appraisals of job characteristics as challenges and/or hindrances, and examined how these job characteristics and their appraisals interacted in affecting employee well-being across two studies involving 514 employees from multiple organizations and a sample of 314 nurses from a single hospital, respectively. Overall, our results supported the notion that the appraisals of job characteristics as challenges and hindrances are not mutually exclusive. The job characteristics that are normally categorized as job demands and job resources could be appraised as challenges and hindrances simultaneously.

In addition, the appraisals of job demands and resources could moderate some of the relationships between demands/resources and well-being in terms of employee engagement and burnout. Specifically, the more an employee perceives a certain job demand (i.e., time urgency, role conflict, or emotional demand) to be challenging, the weaker the relationship between this job demand and employee engagement/burnout. Further, the more the employee perceives a certain job resource to be challenging, the stronger the relationship between this resource and employee engagement/burnout. Conversely, if an employee perceives a basically favorable situation (i.e., autonomy, supervisor and colleague support, and feedback) more as a hindrance, the positive relationships between job resources and engagement and the negative relationships between resources and burnout are weaker. The findings of the current study suggest that job characteristics have a particular basic valence (i.e., that of a job demand vs. a job resource, cf. Demerouti et al., 2001; or that of a challenge vs. a hindrance, cf. LePine et al., 2016), and that individual appraisal of these characteristics plays an essential role in the effects of these characteristics on employee well-being. In particular, the appraisals that are incongruent with the basic valence of a job characteristic yields a more salient impact on employee well-being (i.e., appraising job demands as challenging, or appraising job resources as hindering), as shown in the present study. Although the results across the two studies were not exactly the same (for a comparison of these two studies, see Table 9), the overall interaction patterns obtained in both studies are in line with our hypotheses. These inconsistent findings might have occurred for two empirical reasons. The first relates to the different sampling methods. Study 1 used employees from multiple organizations, whereas Study 2 used employees in a single hospital. Bakker and Sanz-Vergel (2013) found that emotional demands were appraised as challenges by nurses, and they suggested that whether job demands act as a challenge or a hindrance varies across occupations and individuals. Alternatively, the differences between both studies might be because of different measurements. As aforementioned, in Study 2 we asked employees to appraise their *current* job characteristics, whereas Study 1 measured employees’ *general* appraisals.

**TABLE 9:** A comparison of the interaction effect between job characteristics and appraisals among two studies.

**Hypotheses**	**Hypothesized Relationships**	**Study 1**	**Study 2**
Direct effect	H1: time urgency, role conflict and emotional demands will be positively related to burnout and negatively to engagement	Yes 6/6	Yes 6/6
Demands* CA ON Engagement	H2: the negative relation between demands and engagement is weaker when challenge appraisal is high	Yes 3/3	No 0/3
Demands* CA ON Burnout	H3: the positive relation between demands and burnout is weaker when challenge appraisal is high	No 0/3	Partial 2/3
Demands* HA ON Engagement	H4: the negative relation between demands and engagement is stronger when hindrance appraisal is high	No 0/3	No 0/3
Demands* HA ON Burnout	H5: the positive relation between demands and burnout is stronger when hindrance appraisal is high	Partial 2/3	No 0/3
Direct effect	H6: job resources will be positively related to work engagement and negatively to burnout	Yes 8/8	Yes 8/8
Resources* CA ON Engagement	H7: the positive relation between resources and engagement is stronger when challenge appraisal is high	No 0/4	Yes 4/4
Resources* CA ON Burnout	H8: the negative relation between resources and burnout is stronger when challenge appraisal is high	No 0/4	Partial 1/4
Resources* HA ON Engagement	H9: the positive relation between resources and engagement is weaker when hindrance appraisal is high	Partial 3/4	No 0/4
Resources* HA ON Burnout	H10: the negative relation between resources and burnout is weaker when hindrance appraisal is high	Yes 4/4	No 0/4

*CA, challenge appraisal; HA, hindrance appraisal; Demands include time urgency, role conflict, and emotional demands; Resources include autonomy, supervisor support, colleague support, and feedback.*

### Theoretical Implications

Our study has several theoretical implications. First, this study contributes to the literature on job characteristics theory (e.g., the JD-C model, Karasek, 1979; the JD-R model, Demerouti et al., 2001) and the Challenge-Hindrance Stressor Framework (Cavanaugh et al., 2000) by showing how individuals can appraise job characteristics differentially. Previous research often *a priori* classified job characteristics as either demands or resources (or as challenges vs. hindrances), while ignoring the role of employees’ subjective appraisals of these characteristics (Ohly and Fritz, 2010; Webster et al., 2011; Parker, 2014; González-Morales and Neves, 2015, for notable exceptions). Our results did not find any presumed positive effects for *a priori* “challenge stressors” on employee outcomes (e.g., time pressure), which is in line with a recent meta-analysis (Mazzola and Disselhorst, 2019). Empirical studies also showed that time pressure is negatively related to work engagement (e.g., Baethge et al., 2019; Gabriel et al., 2019; Kronenwett and Rigotti, 2019). This suggests that the challenge-hindrance stressor model may not be as effective in all contexts as some researchers suggested (e.g., O’Brien and Beehr, 2019). Our study drew upon appraisal theory (lazarus and Folkman, 1984) and tested empirically whether job characteristics (i.e., normally called “job demands and job resources”) can be simultaneously appraised as challenges and hindrances. We demonstrated that specific job characteristics can be appraised as being both a challenge *and* a hindrance simultaneously. Specifically, Study 1 found that among three selected job demands, time urgency was primarily appraised as a challenge, and was to some degree also appraised as a hindrance. Role conflict and emotional demands were more likely to be appraised as hindrances, and to some extent as challenges. In Study 2, these job demands were more likely appraised as challenges by nurses and to some degree as hindrances.

These results are largely consistent with Webster et al. (2011), who reported that job demands (e.g., workload, role ambiguity) can simultaneously be perceived as challenges and hindrances to varying degrees. Our findings add to previous studies (e.g., LePine et al., 2005) by suggesting that job demands may not simply be *a priori* categorized as challenges or hindrances. Interestingly, across two studies, we found that time urgency was more likely to be considered as a challenge than a hindrance (similar to role conflict and emotional demands in Study 2); however, it demonstrated a negative effect on work engagement. We argue that when job demands unfold their challenging potential on employee well-being may depend on some boundary conditions. This is consistent with the findings by Kronenwett and Rigotti (2019) who found that time pressure and emotional demands had positive indirect effects on work engagement through task-related achievement when unnecessary tasks are less frequent. Similarly, Baethge et al. (2019) found that time pressure positively related to work engagement only when employees do not work longer. Taken together, our results resonate with these previous research findings by suggesting that whether job demands have challenging or hindering effects may depend on some boundary conditions.

Moreover, job resources may also be experienced differently by employees. Based on appraisal theory (lazarus and Folkman, 1984), we examined how employees appraise their job resources. For four job resources (i.e., autonomy, colleague and supervisor support, feedback from others), we consistently found that employees appraised these resources primarily as challenges and to some degree also as hindrances across two studies. Further, the results showed that challenge appraisals and hindrance appraisals of four resources are negatively correlated among four job resources. These results are in line with the person-job fit theory (Edwards, 1991; van Vianen, 2018) and Warr’s (1987) vitamin model, which proposed that job resources are not always desirable for all employees. In summary, our findings extend the job characteristics literature by revealing that employees can experience job characteristics concurrently as challenges and hindrances, and that hindrance appraisal can inhibit the positive effect of job resources on employee well-being.

Third, we examined the moderating role of appraisals on the relationship between job characteristics and employee well-being. By doing so, we advance the literature by suggesting how cognitive appraisals influence employee well-being and revealing the boundary conditions of the job characteristics–employee well-being relationship. While some studies have examined the mediating role of appraisals (e.g., Boswell et al., 2004; Liu and Li, 2018), relatively less attention has been paid to the moderating role of appraisals in the job characteristics literature (O’Brien and Beehr, 2019). Our study addressed this limitation and showed that challenge appraisals moderate the associations between time urgency, role conflict, and emotional demands and work engagement, which resonates with the findings of a recent study (Li et al., 2020). Similarly, hindrance appraisals moderate the relationship between job demands (time urgency and emotional demands) and burnout as found in Study 1. Koopmann et al. (2018) found that reappraisal can help prevention-focused employees to reframe their negative perceptions of events to be more neutral, thereby experiencing less negative emotions. These findings are consistent with Wortman and Silver’s (1989) review that people who discover something positive in a negative situation show less distress than those who do not (e.g., Natterson and Knudson, 1960; Folkman, 1984).

### Limitations and Directions for Future Research

Our research is not without several limitations. First, we used a set of scenarios describing hypothesized situations instead of referring to participants’ actual jobs, to measure the appraisals of the job characteristics in Study 1. As a result, these appraisals may reflect a general belief rather than measure participants’ appraisals of the characteristics of their own jobs. This limitation was reduced by measuring appraisal in a different approach (i.e., referring to employees’ current job characteristics instead of referring to a hypothetical situation) and using employees with similar job characteristics (i.e., nurses) in Study 2.

Second, to maximize the retention rates of our sample and guarantee adequate statistical power, we utilized a cross-sectional design; therefore, some concerns exist regarding common method bias (Podsakoff et al., 2003). However, we strived to reduce this issue by (a) conducting a replication study; (b) performing CFA, which showed that our focal variables can be differentiated from each other; and (c) an additional unmeasured common method factor that was included in our CFA model explained less than 10% of the variance in the items, supporting that common method bias does not have a substantial impact on the present findings. In addition, our hypothesized relationships are consistent with previous studies (Li et al., 2020), and the moderation effect was less likely to be affected by common method bias (Podsakoff et al., 2012; Mitchell et al., 2019); moreover, researchers have suggested that self-report data are valid when examining perceptual outcomes (Chan, 2009), and a meta-analysis has shown that collecting sensitive concepts data from the focal source is more accurate than other-reports (Carpenter et al., 2017). Thus, we believe the results were not unduly influenced by common method bias. Yet, it would be desirable for future research to collect data from other sources as well (e.g., from colleagues), to temporally separate the measurement of these variables, or to include objective measures (e.g., objective job demands, such as overtime working hours or the number of patients to be taken care of, cf. Dwyer and Ganster, 1991) to replicate our findings.

Finally, it would also be fruitful for future research to replicate our findings using more advanced designs like experience sampling methods (Bolger et al., 2003), to see how employees appraise different job characteristics in their daily work. Such research will be able to capture the dynamic interplay of job characteristics, work outcomes and appraisals. The transactional theory of stress (lazarus and Folkman, 1984) denotes that an individual and his/her environment are in a dynamic and constantly changing relationship; this relationship is bidirectional, with both the person and the environment being able to influence the other (Folkman, 1984). To examine this dynamic process, more advanced study designs are needed.

### Implications for Practice

Although with the above limitations, the present study carries several practical implications. First, our study suggests that employees benefit from viewing a demanding situation as a challenge, i.e., as an opportunity for gain and growth, because it can buffer the detrimental effects of job demands on employees’ well-being. Note that this does not mean we want to trivialize the effects of work stress nor do we want to suggest that victims of work stress should blame themselves; we do want to emphasize that good job design is still the best way to prevent work stress (Grant and Parker, 2009). However, at the same time, it should be noted that workers can to some degree improve the characteristics of their jobs (e.g., change their cognitions or behaviors through job crafting, Tims et al., 2013), meaning that they do not necessarily need to be the passive recipients of the adverse influence of a poorly designed job. Importantly, managers should provide a good job design and a work climate in which it is feasible and reasonable for employees to appraise work demands as challenging (e.g., adopting positive leadership styles; LePine et al., 2016). Managers may use training programs to develop employees’ cognitive appraisals to help them see the potential opportunities in a demanding situation and reduce their levels of work stress. A meta-analysis has shown that cognitive-behavioral interventions (which aim to change an individual’s appraisal and their responses) consistently provide more positive effects than other stress management interventions in work settings (Richardson and Rothstein, 2008). Thus, managers may consider adopting such interventions within the organizations.

In addition, managers should establish a more balanced view that not all resources are equally beneficial for all employees since employees may appraise these resources differently. Our research shows that the more an employee perceives a certain job resource to be challenging, the stronger the relationship between this resource and employee engagement/burnout. However, if an employee perceives job resources more as hindrances, the positive relationships between job resources and engagement and the negative relationships between resources and burnout are weaker. Thus, managers need to help employees (especially those who seeing job resources as hindering) by creating a working environment that promotes employees’ challenge appraisals of job resources and avoiding perceiving job resources as hindering. In summary, managers and employees can work together to help employees create a healthy working life.

## Conclusion

How do employees evaluate their job characteristics? Our study showed that they perceive job characteristics differently and appraise them both as challenges and hindrances. In addition, such appraisals can alter the relationship between job demands/resources and employee well-being in terms of burnout and engagement. In particular, when employees see job demands as a challenge (i.e., seeing something bad as good), the adverse effect of job demands on engagement and burnout were weaker; when they consider job resources as a hindrance (i.e., seeing something good as bad) weakens the beneficial effect of job resources on employee work engagement and burnout. But a positive interpretation (challenge appraisal) of job resources will strengthen its positive effect on employee engagement and burnout. This knowledge is important in understanding how job characteristics influence employees and in guiding effective stress management efforts.

## Data Availability Statement

The data that support the findings of this study are available from the corresponding author upon reasonable request.

## Ethics Statement

Ethical review and approval was not required for the study on human participants in accordance with the local legislation and institutional requirements. Participants provided their written informed consent to participate in this study.

## Author Contributions

PL, TT, MP, and YZ: conceptualization and writing – review and editing. PL: data curation, formal analysis, investigation, methodology, project administration, software, validation, visualization, and writing – original draft. TT and MP: project administration and supervision. YZ: investigation, methodology, formal analysis of Study 2, and resources. All authors contributed to the article and approved the submitted version.

## Conflict of Interest

The authors declare that the research was conducted in the absence of any commercial or financial relationships that could be construed as a potential conflict of interest.

## Publisher’s Note

All claims expressed in this article are solely those of the authors and do not necessarily represent those of their affiliated organizations, or those of the publisher, the editors and the reviewers. Any product that may be evaluated in this article, or claim that may be made by its manufacturer, is not guaranteed or endorsed by the publisher.
