# Supplementary material for: In the Eye of the Beholder: Challenge and Hindrance Appraisals of Work Characteristics and Their Implications for Employee’s Well-Being
Source: Front Psychol. 2021 Sep 7;12:708309. doi: 10.3389/fpsyg.2021.708309 (PMC8452854; doi:10.3389/fpsyg.2021.708309)
Supplement: Supplementary file 1 [file Data_Sheet_1.pdf]

Supplementary Materials

TABLE S1. Regression results for the moderation of appraisals on the relationships between job demands and work engagement/Burnout in Study 1 (with control variables).

TABLE S2. Regression results for the moderation of appraisals on the relationships between job resources and work engagement/Burnout in Study 1 ((with control variables).

TABLE S3. Regression results for the moderation of appraisals on the relationships between job demands and work engagement/burnout in Study 2 (with control variables).

TABLE S4. Regression results for the moderation of appraisals on the relationships between job resources and work engagement/burnout in Study 2 (with control variables.

Figures

FIGURE S1. The interaction between time urgency and hindrance appraisal on burnout in Study 1.

FIGURE S2. The interaction between role conflict and challenge appraisal on engagement in Study 1.

FIGURE S3. The interaction between emotional demands and challenge appraisal on engagement in Study 1.

FIGURE S4. The interaction between autonomy and hindrance appraisal on burnout in Study 1.

FIGURE S5. The interaction between autonomy and hindrance appraisal on engagement in Study 1.

FIGURE S6. The interaction between feedback and hindrance appraisal on burnout in Study 1.

FIGURE S7. The interaction between feedback and hindrance appraisal on engagement in Study 1.

FIGURE S8. The interaction between colleague support and hindrance appraisal on burnout in Study 1.

FIGURE S9. The interaction between colleague support and hindrance appraisal on engagement in Study 1.

FIGURE S10. The interaction between emotional demands and challenge appraisal on burnout in Study 2.

FIGURE S11. The interaction between autonomy and challenge appraisal on engagement in Study 2.

FIGURE S12. The interaction between supervisor support and challenge appraisal on engagement in Study 2.

FIGURE S13. The interaction between colleague support and challenge appraisal on engagement in Study 2.

TABLE S1. Regression results for the moderation of appraisals on the relationships between job demands and work engagement/Burnout in Study 1 (with control variables).

	Step 1				*Step 2*				*Step 3*			
	**Burnout**		**Engagement**		**Burnout**		**Engagement**		**Burnout**		**Engagement**	
**Predictors**	**Beta**	***p***	**Beta**	***p***	**Beta**	***p***	**Beta**	***p***	**Beta**	***p***	**Beta**	***p***
Gender	-0.04	0.33	-0.03	0.51	-0.02	0.56	-0.03	0.44	-0.04	0.38	-0.02	0.63
Age	-0.04	0.56	-0.01	0.93	-0.06	0.29	0.00	0.99	-0.05	0.33	-0.01	0.92
Education	-0.01	0.76	0.13	0.00	-0.08	0.05	0.13	0.00	-0.08	0.04	0.13	0.00
Work time	0.04	0.36	-0.19	0.00	-0.01	0.77	-0.12	0.01	-0.01	0.83	-0.12	0.00
Tenure	-0.03	0.62	0.07	0.21	-0.03	0.63	0.05	0.40	-0.04	0.47	0.06	0.28
Industry	-0.06	0.17	-0.07	0.10	-0.02	0.65	-0.08	0.05	-0.02	0.65	-0.08	0.05
Time Urgency					0.42	0.00	-0.21	0.00	0.42	0.00	-0.21	0.00
Challenge Appraisals					-0.01	0.76	0.28	0.00	-0.03	0.50	0.30	0.00
Hindrance Appraisals					0.14	0.00	0.10	0.04	**0.15****	**0.00**	**0.09**	**0.06**
Time Urgency × CA									**-0.07**	**0.14**$^{*\backslash}$	**0.10**	**0.04**
Time Urgency × HA									0.11	0.02	-0.08	0.11
R^2	0.01		0.07		0.21		0.15		0.24		0.17	
Gender	-0.04	0.33	-0.03	0.51	-0.03	0.47	-0.04	0.34	-0.03	0.43	-0.04	0.38
Age	-0.04	0.56	-0.01	0.93	-0.01	0.86	-0.04	0.45	-0.01	0.89	-0.05	0.39
Education	-0.01	0.76	0.13	0.00	-0.05	0.23	0.12	0.00	-0.05	0.20	0.13	0.00
Work time	0.04	0.36	-0.19	0.00	0.03	0.44	-0.16	0.00	0.03	0.49	-0.15	0.00
Tenure	-0.03	0.62	0.07	0.21	0.00	0.97	0.04	0.45	-0.01	0.92	0.05	0.38
Industry	-0.06	0.17	-0.07	0.10	-0.05	0.24	-0.07	0.11	-0.05	0.26	-0.07	0.10
Role Conflict					0.45	0.00	-0.24	0.00	0.44	0.00	-0.23	0.00
Challenge Appraisals					-0.04	0.41	0.25	0.00	-0.04	0.35	0.26	0.00
Hindrance Appraisals					0.18	0.00	0.01	0.90	0.19	0.00	0.00	0.98
Role Conflict × CA									**-0.03**	**0.55**	**0.13****	**0.01**
Role Conflict × HA									**0.05**	**0.28**	**0.02**	**0.64**
R^2	0.01		0.07		0.25		0.16		0.26		0.17	
Gender	-0.04	0.33	-0.03	0.51	-0.09	0.02	0.01	0.76	-0.09	0.02	0.02	0.71
Age	-0.04	0.56	-0.01	0.93	-0.04	0.38	-0.01	0.86	-0.05	0.32	-0.01	0.84
Education	-0.01	0.76	0.13	0.00	-0.02	0.54	0.11	0.01	-0.02	0.58	0.10	0.01
Work time	0.04	0.36	-0.19	0.00	0.00	0.91	-0.12	0.00	0.00	0.94	-0.11	0.01
Tenure	-0.03	0.62	0.07	0.21	0.02	0.66	0.02	0.77	0.02	0.73	0.03	0.62
Industry	-0.06	0.17	-0.07	0.10	-0.02	0.67	-0.09	0.03	-0.01	0.86	-0.09	0.02
Emotional Demands					0.59	0.00	-0.33	0.00	0.60	0.00	-0.34	0.00
Challenge Appraisals					-0.04	0.35	0.32	0.00	-0.05	0.24	0.33	0.00
Hindrance Appraisals					0.03	0.51	0.14	0.00	0.05	0.28	0.11	0.02
Emotional Demands × CA									**0.01**	**0.87**	**0.10***	**0.04**
Emotional Demands × HA									**0.12***	**0.01**	**-0.08**	**0.10**
R^2	0.01		0.07		0.36		0.22		0.38		0.25	

Note: $^{*}p < .05$, $^{**}p < .01$, $^{***}p < .001$. CA = challenge appraisal; HA = hindrance appraisal. Standardized regression coefficients were reported. Gender (1 =male, 2 = female).

TABLE S2. Regression results for the moderation of appraisals on the relationships between job resources and work engagement/Burnout in Study 1 ((with control variables).

	Step 1				***Step 2***				***Step 3***			
	**Burnout**		**Engagement**		**Burnout**		**Engagement**		**Burnout**		**Engagement**	
**Predictors**	**Beta**	***p***	**Beta**	***p***	**Beta**	***p***	**Beta**	***p***	**Beta**	***p***	**Beta**	***p***
Gender	-.04	.33	-.03	.51	-.01	.86	-.05	.18	-.01	.82	-.05	.19
Age	-.04	.56	-.01	.93	-.05	.35	.00	.96	-.05	.33	.00	.94
Education	-.01	.76	.13	.00	.02	.67	.07	.08	.02	.71	.07	.07
Work time	.04	.36	-.19	.00	.06	.13	-.13	.00	.06	.17	-.12	.00
Tenure	-.03	.62	.07	.21	-.03	.55	.06	.27	-.03	.53	.06	.26
Industry	-.06	.17	-.07	.10	-.03	.48	-.08	.06	-.04	.40	-.07	.07
Autonomy					-.35	.00	.35	.00	-.31	.00	.31	.00
Challenge Appraisals					-.08	.10	.23	.00	-.08	.12	.23	.00
Hindrance Appraisals					.21	.00	.20	.00	.13	.03	.26	.00
Autonomy× CA									**-.04**	**.43**	**.06**	**.21**
Autonomy × HA									**.12***	**.03**	**-.10**	**.06**
R^2	.11		.07		.19		.25		.20		.26	
Gender	-.04	.33	-.03	.51	-.04	.38	-.03	.42	-.03	.43	-.03	.38
Age	-.04	.56	-.01	.93	-.04	.44	.02	.76	-.05	.38	.02	.71
Education	-.01	.76	.13	.00	.01	.85	.09	.02	.01	.84	.09	.02
Work time	.04	.36	-.19	.00	.02	.63	-.11	.01	.03	.45	-.11	.00
Tenure	-.03	.62	.07	.21	-.02	.69	.06	.20	-.03	.58	.07	.15
Industry	-.06	.17	-.07	.10	-.06	.16	-.06	.11	-.06	.17	-.06	.11
Supervisor Support					-.41	.00	.42	.00	-.37	.00	.40	.00
Challenge Appraisals					-.01	.78	.19	.00	-.03	.47	.19	.00
Hindrance Appraisals					.19	.00	.17	.00	.17	.00	.19	.00
Supervisor Support × CA									**-.06**	**.19**	**.00**	**.93**
Supervisor Support × HA									**.16****	**.00**	**-.15****	**.00**
R^2	.01		.07		.23		.30		.27		.32	
Gender	-.04	.33	-.03	.51	-.04	.32	-.02	.69	-.04	.31	-.02	.70
Age	-.04	.56	-.01	.93	-.05	.37	.03	.62	-.06	.25	.04	.48
Education	-.01	.76	.13	.00	.00	.94	.11	.01	-.01	.80	.12	.00
Work time	.04	.36	-.19	.00	.06	.18	-.15	.00	.06	.16	-.15	.00
Tenure	-.03	.62	.07	.21	-.03	.60	.05	.38	-.03	.62	.05	.38
Industry	-.06	.17	-.07	.10	-.04	.32	-.07	.11	-.04	.38	-.07	.08
Colleague Support					-.32	.00	.33	.00	-.29	.00	.30	.00
Challenge Appraisals					-.03	.55	.16	.00	-.03	.54	.17	.00
Hindrance Appraisals					.20	.00	.12	.01	.20	.00	.12	.01
Colleague Support × CA									**-.01**	**.87**	**.04**	**.39**
Colleague Support × HA									**.15****	**.00**	**-.13****	**.00**
R^2	.01		.07		.18		.21		.20		.24	

Continued

Gender	-.04	.33	-.03	.51	-.05	.26	-.01	.87	-.05	.26	-.01	.90
Age	-.04	.56	-.01	.93	-.03	.63	.01	.91	-.03	.58	.01	.89
Education	-.01	.76	.13	.00	.01	.79	.08	.05	.01	.84	.08	.04
Work time	.04	.36	-.19	.00	.08	.05	-.17	.00	.09	.03	-.17	.00
Tenure	-.03	.62	.07	.21	-.04	.46	.04	.39	-.04	.49	.04	.41
Industry	-.06	.17	-.07	.10	-.05	.24	-.05	.20	-.06	.17	-.05	.24
Feedback					-.29	.00	.31	.00	-.25	.00	.28	.00
Challenge Appraisals					-.10	.03	.23	.00	-.12	.01	.24	.00
Hindrance Appraisals					.19	.00	.19	.00	.18	.00	.19	.00
Feedback × CA									**-.07**	**.10**	**.02**	**.57**
Feedback × HA									**.16****	**.00**	**-.11****	**.01**
R^2	.01		.07		.18		.24		.22		.25	

Note: **p* < .05, ***p* < .01, ****p* < .001. CA = challenge appraisal; HA = hindrance appraisal. Standardized regression coefficients were reported. Gender (1 =male, 2 = female).

TABLE S3. Regression results for the moderation of appraisals on the relationships between job demands and work engagement/burnout in Study 2 (with control variables).

	Step 1				*Step 2*				*Step 3*			
	**Burnout**		**Engagement**		**Burnout**		**Engagement**		**Burnout**		**Engagement**	
**Predictors**	**Beta**	*p*	**Beta**	*p*	**Beta**	*p*	**Beta**	*p*	**Beta**	*p*	**Beta**	*p*
Gender	.05	.36	-.01	.86	.04	.34	.00	.98	.04	.37	.00	.95
Age	-.10	.25	.22	.01	-.10	.15	.19	.01	-.09	.18	.19	.01
Education	-.01	.83	.08	.16	-.05	.29	.08	.06	-.04	.39	.09	.05
Work time	.02	.74	.00	.96	-.02	.62	.06	.16	-.03	.52	.06	.15
Tenure	-.07	.45	.01	.89	-.03	.72	-.02	.82	-.03	.61	-.01	.87
Time Urgency					.35	.00	-.13	.01	.35	.00	-.12	.02
Challenge Appraisals					-.21	.00	.39	.00	-.20	.00	.38	.00
Hindrance Appraisals					.27	.00	-.25	.00	.28	.00	-.26	.00
Time Urgency × CA									**-.13***	**.04**	**.04**	**.53**
Time Urgency × HA									**-.04**	**.47**	**.07**	**.22**
R^2	.03		.06		.42		.43		.43		.43	
Gender	.05	.36	-.01	.86	.11	.02	-.07	.21	.11	.03	-.06	.25
Age	-.10	.25	.22	.01	-.03	.68	.15	.07	-.02	.80	.13	.10
Education	-.01	.83	.08	.16	-.03	.59	.08	.10	-.02	.67	.08	.12
Work time	.02	.74	.00	.96	.02	.67	.03	.62	.02	.66	.02	.64
Tenure	-.07	.45	.01	.89	-.04	.63	.00	.96	-.05	.52	.02	.85
Role Conflict					.43	.00	-.22	.00	.43	.00	-.22	.00
Challenge Appraisals					-.11	.07	.27	.00	-.10	.09	.27	.00
Hindrance Appraisals					.19	.00	-.09	.17	.21	.00	-.10	.12
Role Conflict × CA									**-.10**	**.11**	**.08**	**.20**
Role Conflict × HA									**.00**	**.98**	**-.01**	**.90**
R^2	.03		.06		.32		.22		.33		.23	
Gender	.05	.36	-.01	.86	-.02	.72	.02	.62	-.02	.65	.03	.56
Age	-.10	.25	.22	.01	-.08	.21	.16	.02	-.08	.24	.16	.03
Education	-.01	.83	.08	.16	-.03	.54	.07	.11	-.02	.56	.07	.12
Work time	.02	.74	.00	.96	-.01	.75	.05	.23	-.02	.72	.06	.22
Tenure	-.07	.45	.01	.89	-.02	.80	.00	.98	-.04	.56	.02	.81
Emotional Demands					.55	.00	-.37	.00	.53	.00	-.34	.00
Challenge Appraisals					-.19	.00	.41	.00	-.17	.00	.39	.00
Hindrance Appraisals					.12	.02	.01	.92	.14	.01	-.01	.88
Emotional Demands × CA									**-.14***	**.01**	**.12***	**.03**
Emotional Demands × HA									**-.05**	**.30**	**.03**	**.60**
R^2	.03		.06		.47		.38		.48		.39	

Note: CA = challenge appraisal; HA = hindrance appraisal. Standardized regression coefficients were reported.

TABLE S4. Regression results for the moderation of appraisals on the relationships between job resources and work engagement/burnout in Study 2 (with control variables.

	Step 1				***Step 2***				***Step 3***			
	**Burnout**		**Engagement**		**Burnout**		**Engagement**		**Burnout**		**Engagement**	
**Predictors**	**Beta**	***p***	**Beta**	***p***	**Beta**	***p***	**Beta**	***p***	**Beta**	***p***	**Beta**	***p***
Gender	.05	.36	-.01	.86	.07	.19	-.02	.64	.07	.18	-.02	.70
Age	-.10	.25	.22	.01	-.07	.40	.18	.02	-.07	.41	.18	.02
Education	-.01	.83	.08	.16	-.03	.57	.09	.09	-.03	.57	.09	.09
Work time	.02	.74	.00	.96	-.01	.89	.06	.26	-.01	.90	.06	.24
Tenure	-.07	.45	.01	.89	-.04	.59	-.01	.93	-.04	.57	-.01	.91
Autonomy					-.34	.00	.35	.00	-.35	.00	.34	.00
Challenge Appraisals					-.09	.09	.21	.00	-.09	.12	.23	.00
Hindrance Appraisals					.19	.00	-.04	.48	.19	.00	-.04	.42
Autonomy× CA									**.02**	**.67**	**.12***	**.04**
Autonomy × HA									**.00**	**1.00**	**.06**	**.26**
R^2	.03		.06		.25		.27		.25		.28	
Gender	.05	.36	-.01	.86	.06	.26	-.01	.84	.06	.26	.00	.97
Age	-.10	.25	.22	.01	-.12	.12	.23	.00	-.12	.13	.22	.00
Education	-.01	.83	.08	.16	-.02	.70	.08	.08	-.02	.63	.09	.06
Work time	.02	.74	.00	.96	.01	.90	.03	.50	.00	.97	.04	.41
Tenure	-.07	.45	.01	.89	-.03	.74	-.02	.77	-.03	.74	-.03	.67
Supervisor Support					-.34	.00	.38	.00	-.34	.00	.37	.00
Challenge Appraisals					-.19	.00	.27	.00	-.20	.00	.29	.00
Hindrance Appraisals					.16	.00	-.03	.54	.15	.00	-.03	.59
Supervisor Support × CA									**-.01**	**.86**	**.09***	**.05**
Supervisor Support × HA									**.04**	**.48**	**.01**	**.83**
R^2	.03		.06		.28		.36		.28		.36	
Gender	.05	.36	-.01	.86	.03	.58	.02	.73	.03	.60	.02	.68
Age	-.10	.25	.22	.01	-.12	.12	.20	.01	-.12	.12	.22	.00
Education	-.01	.83	.08	.16	.00	.93	.06	.22	-.01	.80	.08	.10
Work time	.02	.74	.00	.96	-.02	.74	.07	.13	-.01	.77	.06	.17
Tenure	-.07	.45	.01	.89	.00	.98	-.02	.78	.00	1.00	-.04	.62
Colleague Support					-.36	.00	.37	.00	-.35	.00	.36	.00
Challenge Appraisals					-.14	.02	.27	.00	-.15	.01	.29	.00
Hindrance Appraisals					.22	.00	-.04	.45	**.22****	**.00**	**-.05**	**.33**
Colleague Support × CA									**-.04**	**.44**	**.16****	**.00**
Colleague Support × HA									.01	.79	.05	.37
R^2	.03		.06		.29		.34		.29		.36	

Note: CA = challenge appraisal; HA = hindrance appraisal. Standardized regression coefficients were reported.

TABLE S4 continued

**Predictors**	**Beta**	***p***	**Beta**	***p***	**Beta**	***p***	**Beta**	***p***	**Beta**	***p***	**Beta**	***p***
Gender	0.05	0.36	-0.01	0.86	0.04	0.39	0.00	1.00	0.06	0.25	-0.01	0.91
Age	-0.10	0.25	0.22	0.01	-0.14	0.09	0.23	0.00	-0.13	0.10	0.23	0.00
Education	-0.01	0.83	0.08	0.16	0.00	0.98	0.05	0.32	-0.02	0.73	0.06	0.22
Work time	0.02	0.74	0.00	0.96	0.03	0.60	0.02	0.65	0.01	0.78	0.03	0.53
Tenure	-0.07	0.45	0.01	0.89	-0.02	0.78	-0.02	0.80	-0.02	0.75	-0.02	0.78
Feedback					-0.25	0.00	0.29	0.00	-0.23	0.00	0.29	0.00
Challenge Appraisals					-0.17	0.00	0.30	0.00	-0.14	0.02	0.27	0.00
Hindrance Appraisals					0.24	0.00	-0.05	0.37	0.26	0.00	-0.08	0.19
Feedback × CA									**-0.22****	**0.00**	**0.18****	**0.00**
Feedback × HA									**-0.03**	**0.63**	**0.08**	**0.20**
R^2	0.03		0.06		0.23		0.28		0.27		0.30	

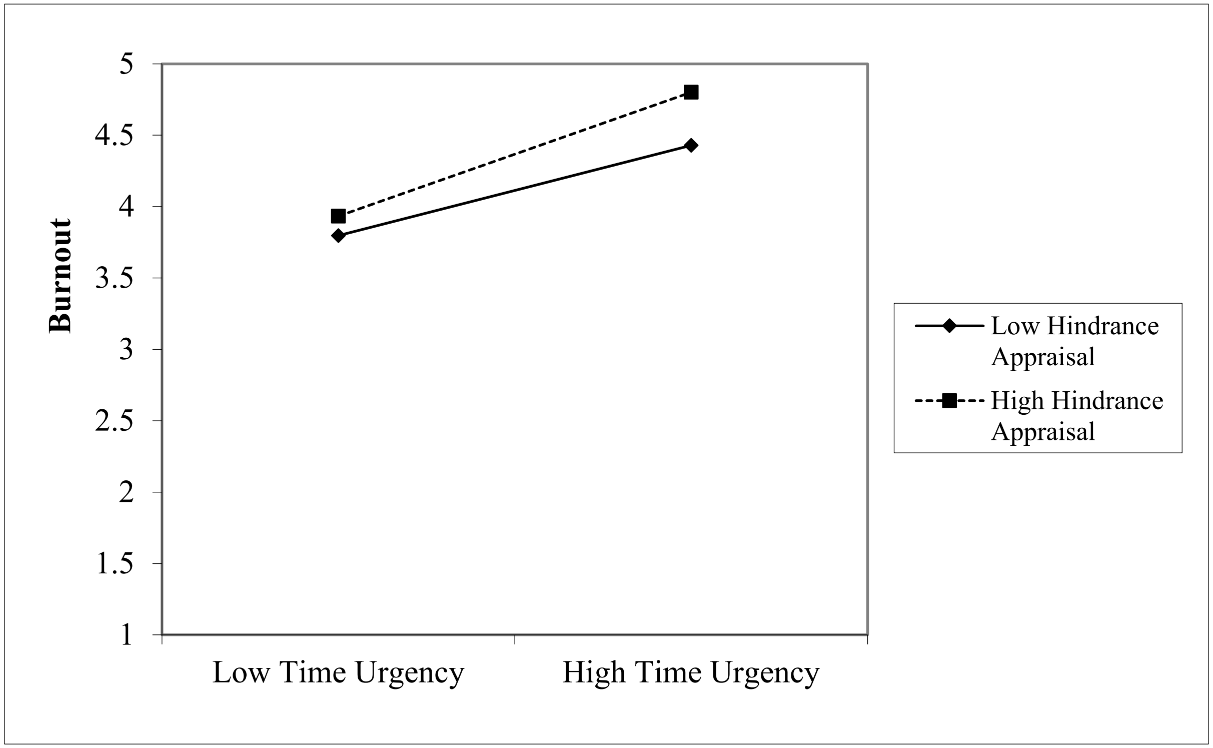

FIGURE S1. The interaction between time urgency and hindrance appraisal on burnout in Study 1.

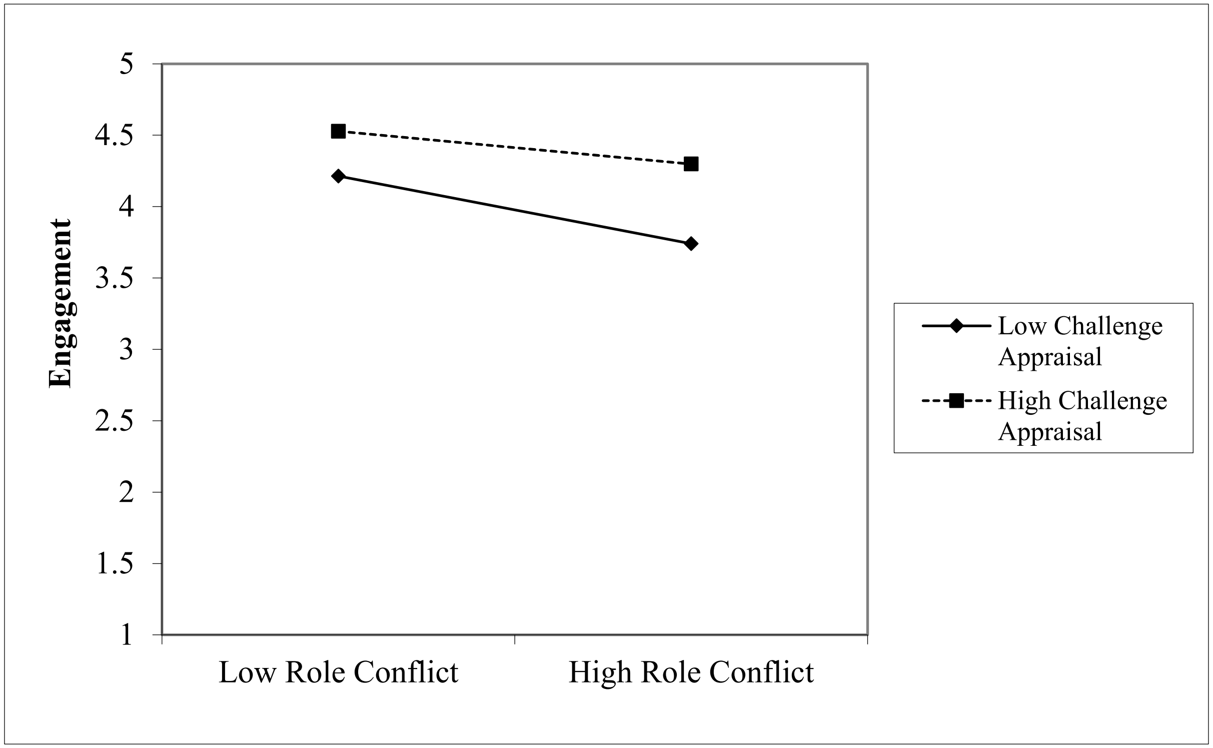

FIGURE S2. The interaction between role conflict and challenge appraisal on engagement in Study 1.

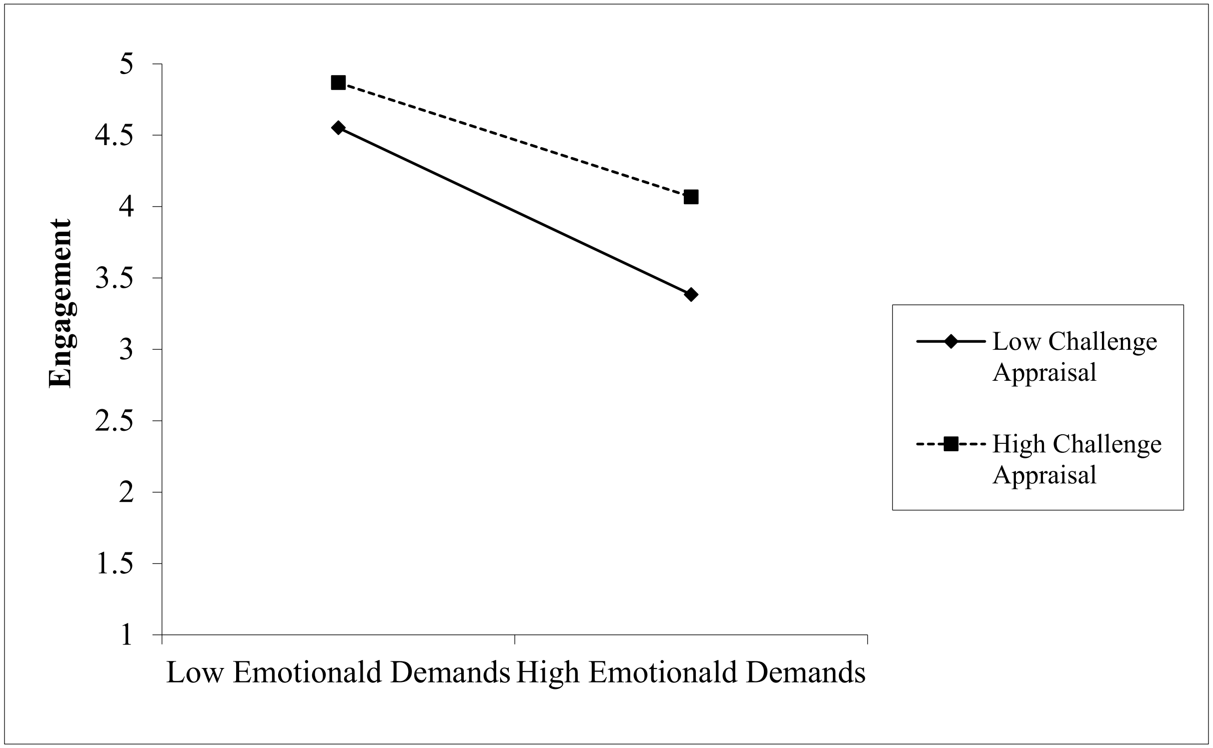

FIGURE S3. The interaction between emotional demands and challenge appraisal on engagement in Study 1.

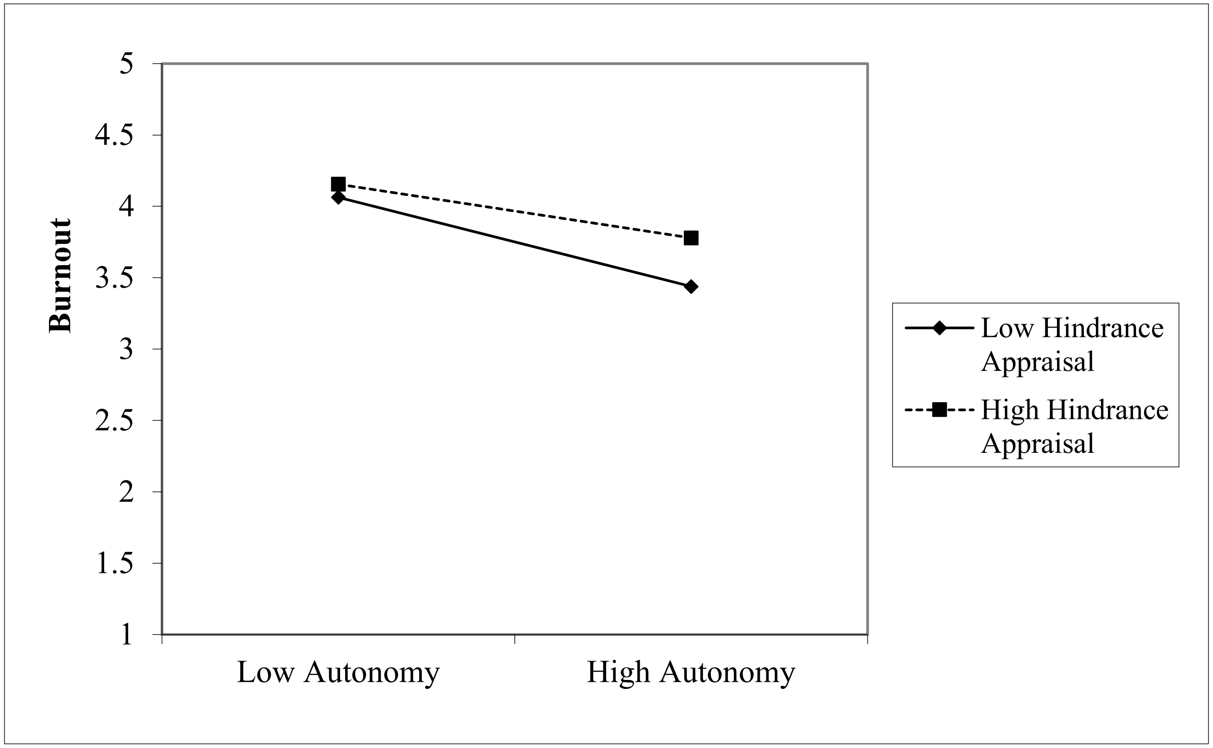

FIGURE S4. The interaction between autonomy and hindrance appraisal on burnout in Study 1.

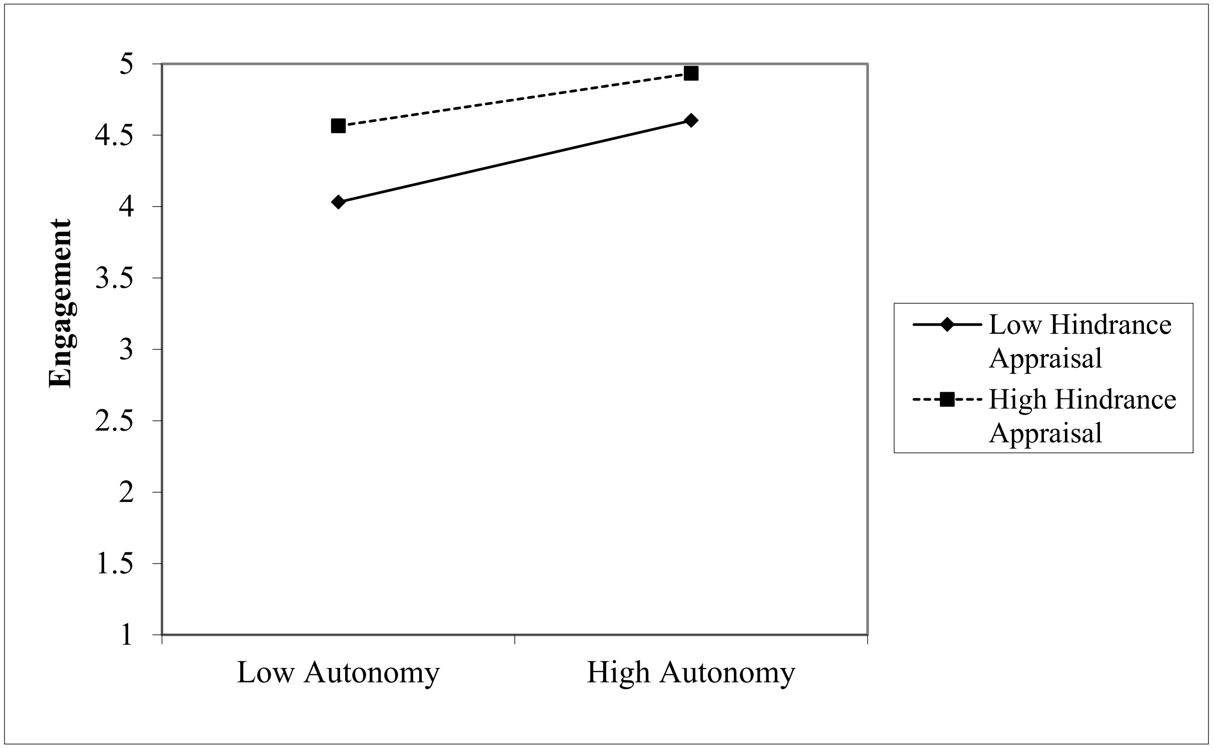

FIGURE S5. The interaction between autonomy and hindrance appraisal on engagement in Study 1.

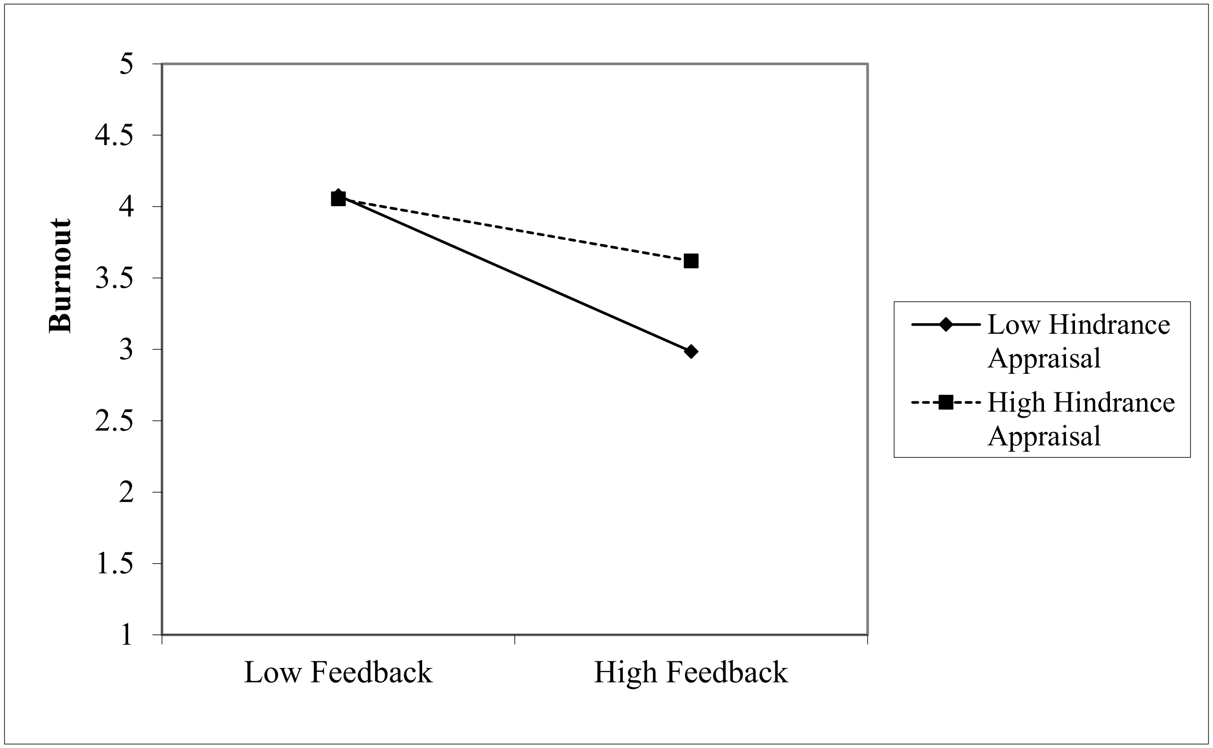

FIGURE S6. The interaction between feedback and hindrance appraisal on burnout in Study 1.

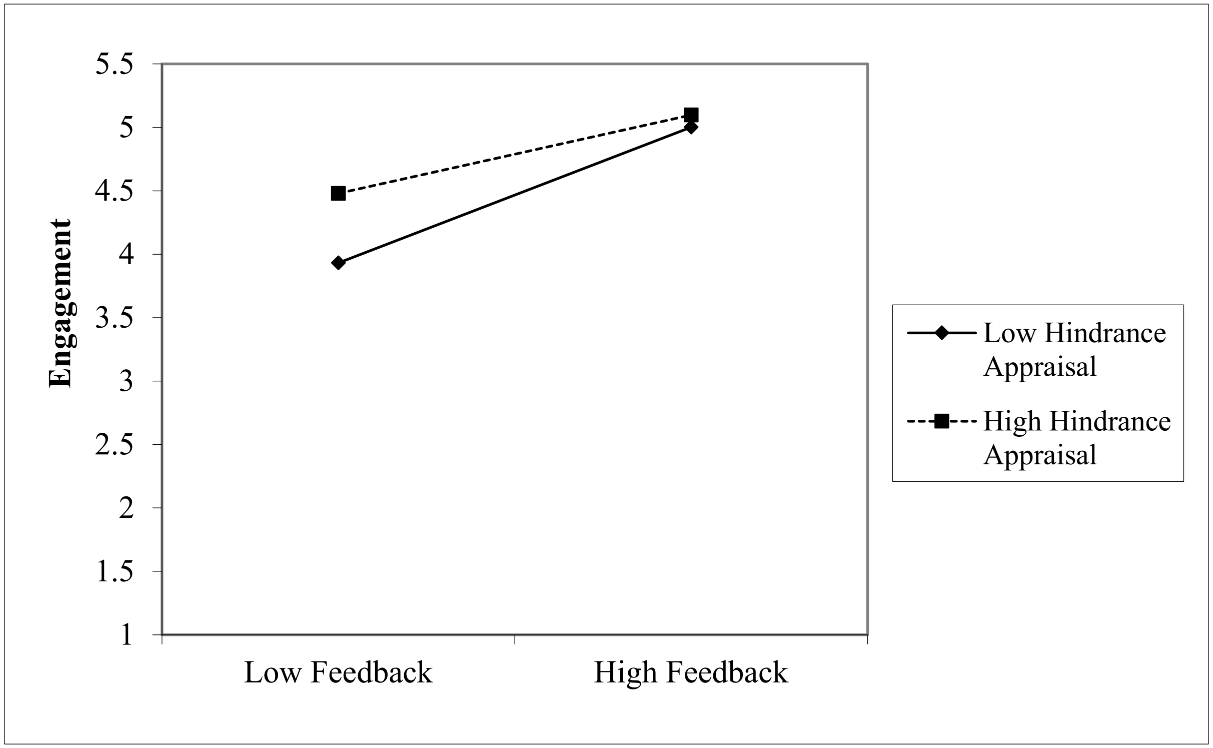

FIGURE S7. The interaction between feedback and hindrance appraisal on engagement in Study 1.

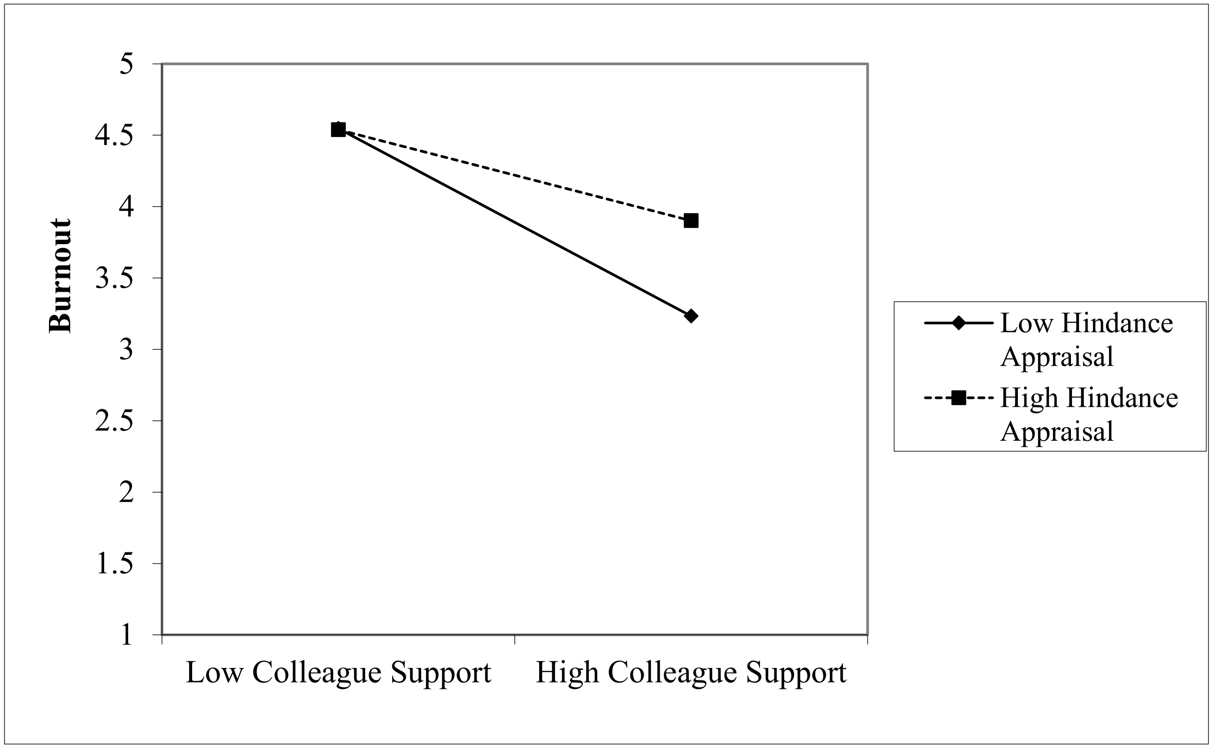

FIGURE S8. The interaction between colleague support and hindrance appraisal on burnout in Study 1.

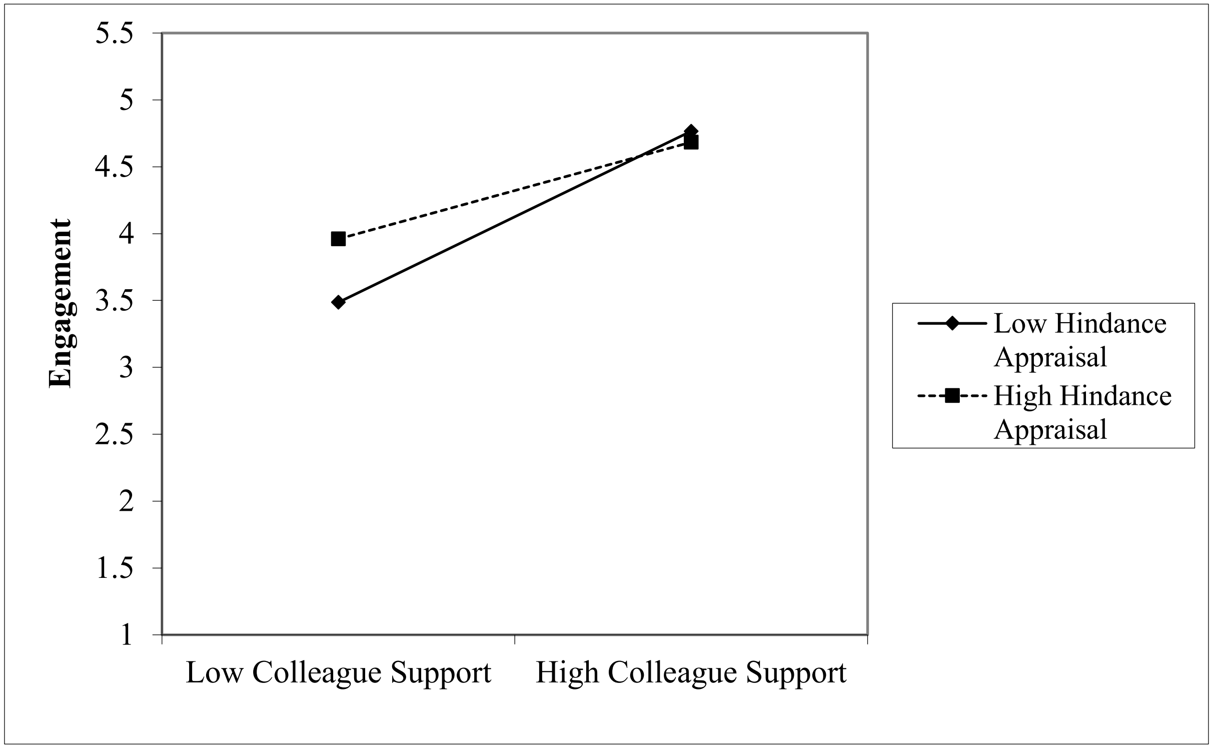

FIGURE S9. The interaction between colleague support and hindrance appraisal on engagement in Study 1.

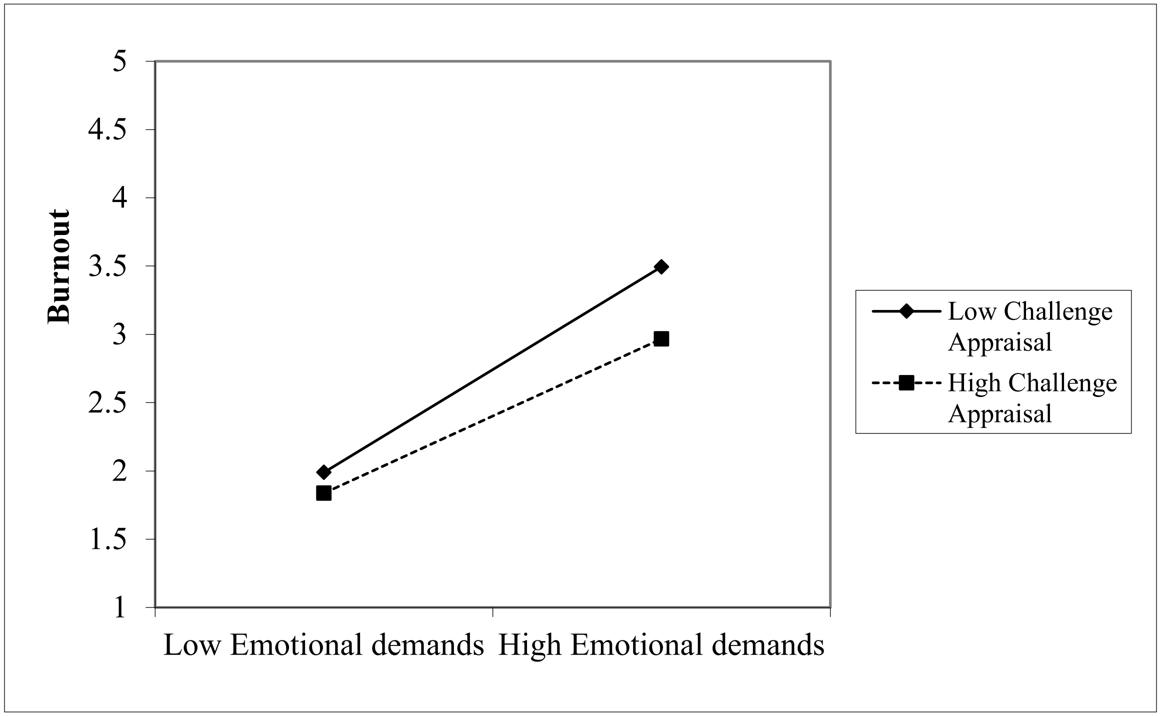

FIGURE S10. The interaction between emotional demands and challenge appraisal on burnout in Study 2.

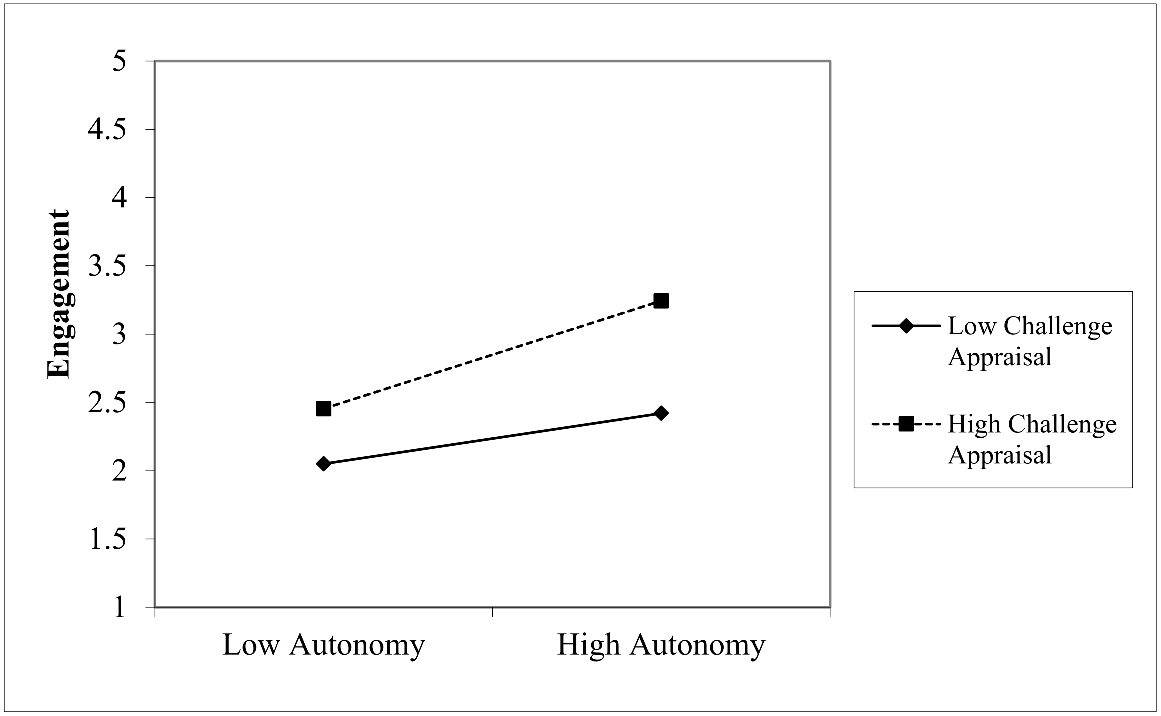

FIGURE S11. The interaction between autonomy and challenge appraisal on engagement in Study 2.

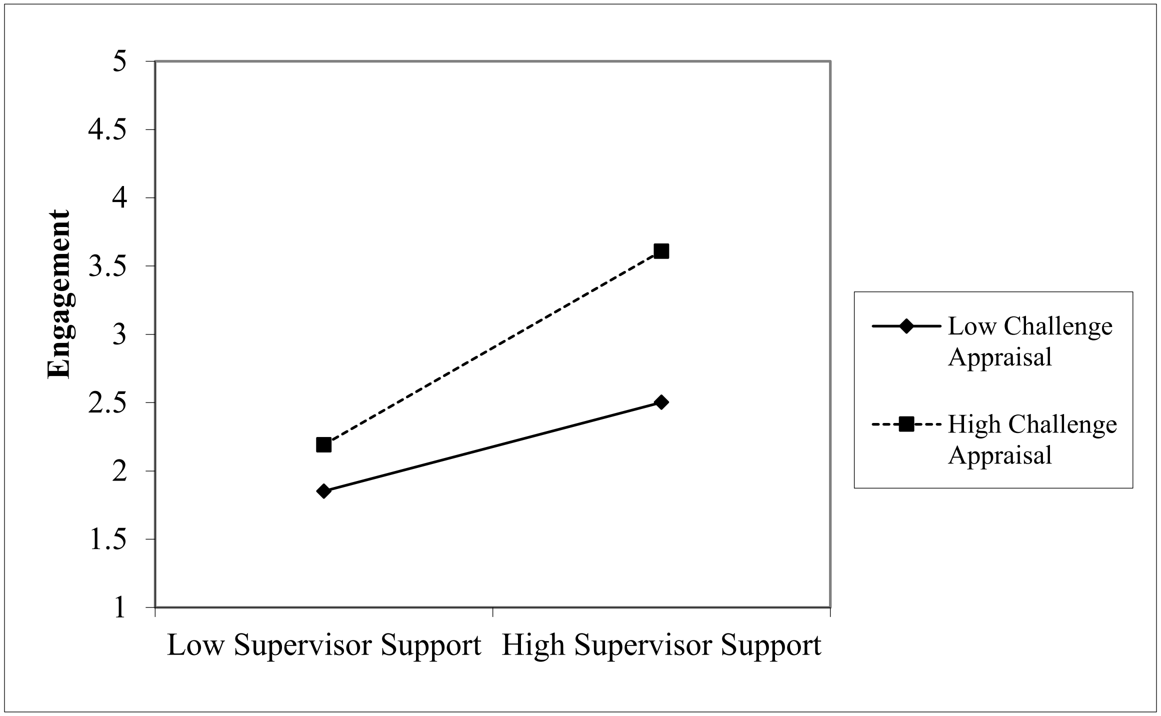

FIGURE S12. The interaction between supervisor support and challenge appraisal on engagement in Study 2.

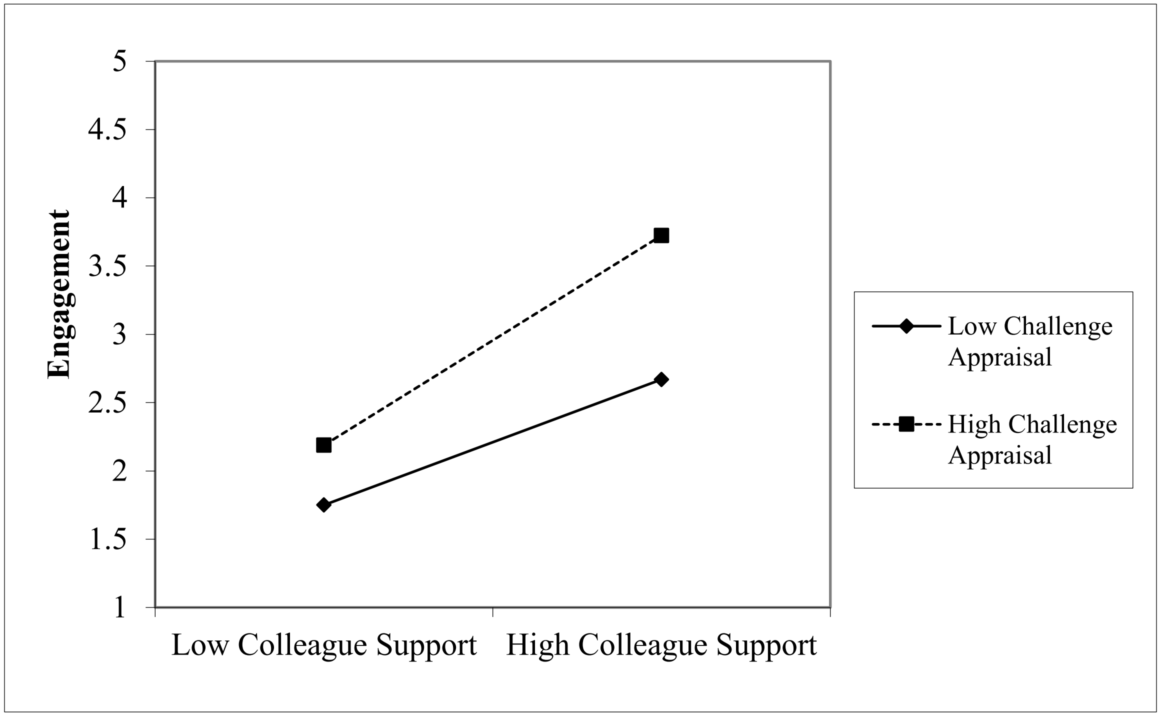

FIGURE S13. The interaction between colleague support and challenge appraisal on engagement in Study 2.
